# Complex organizational structure of the genome revealed by genome-wide analysis of single and alternative promoters in *Drosophila melanogaster*

**DOI:** 10.1186/1471-2164-10-9

**Published:** 2009-01-07

**Authors:** Qianqian Zhu, Marc S Halfon

**Affiliations:** 1 Department of Biochemistry, Buffalo, NY 14214, USA; 2 Department of Biostatistics, Buffalo, NY 14214, USA; 3 Department of Biological Sciences, State University of New York at Buffalo, Buffalo, NY 14214, USA; 4 New York State Center of Excellence in Bioinformatics and the Life Sciences, Buffalo, NY 14203, USA; 5 Department of Molecular and Cellular Biology, Roswell Park Cancer Institute, Buffalo, NY 14263, USA

## Abstract

**Background:**

The promoter is a critical necessary transcriptional *cis*-regulatory element. In addition to its role as an assembly site for the basal transcriptional apparatus, the promoter plays a key part in mediating temporal and spatial aspects of gene expression through differential binding of transcription factors and selective interaction with distal enhancers. Although many genes have multiple promoters, little attention has been focused on how these relate to one another; nor has much study been directed at relationships between promoters of adjacent genes.

**Results:**

We have undertaken a systematic investigation of *Drosophila *promoters. We divided promoters into three groups: unique promoters, first alternative promoters (the most 5' of a gene's multiple promoters), and downstream alternative promoters (the remaining alternative promoters 3' to the first). We observed distinct nucleotide distribution and sequence motif preferences among these three classes. We also investigated the promoters of neighboring genes and found that a greater than expected number of adjacent genes have similar sequence motif profiles, which may allow the genes to be regulated in a coordinated fashion. Consistent with this, there is a positive correlation between similar promoter motifs and related gene expression profiles for these genes.

**Conclusions:**

Our results suggest that different regulatory mechanisms may apply to each of the three promoter classes, and provide a mechanism for "gene expression neighborhoods," local clusters of co-expressed genes. As a whole, our data reveal an unexpected complexity of genomic organization at the promoter level with respect to both alternative and neighboring promoters.

## Background

Coordinated regulation of gene expression is a fundamental process that depends on the binding of transcription factors to a gene's *cis*-regulatory sequences. Absolutely required for transcription initiation of metazoan protein-coding genes is the core promoter, the region of DNA 35–40 bp upstream and downstream of the transcription start site (TSS) [1]. The core promoter contains sequence elements, referred to as "core promoter motifs," which interact with the basal transcription machinery, including RNA polymerase II and the TFIID complex [2]. In recent years, it has become clear that the core promoter, rather than playing a passive role in the spatial and temporal regulation of gene expression, is an important active partner in these events [3,4]. For instance, different promoter sequences are found preferentially associated with certain functional classes of genes, with genes expressed at particular developmental stages, and with genes expressed in the germ line versus the soma [5-8]. Various tissue-specific members of the TATA box-binding protein (TBP) family, such as the TBP-related factors (TRFs), bind preferentially to certain core promoters [4]. There is also substantial evidence for preferred or specific promoter-enhancer interactions, whereby a distal *cis*-regulatory module (CRM, or "enhancer") can stimulate activity from one promoter, but not another [9,10].

A number of mechanisms have been demonstrated to restrict the activity of a CRM to a particular promoter, including insulator elements [11], insulator-bypass or promoter targeting elements [12,13], short-range repression [14], chromatin-mediated silencing [11,15], and preferential interaction with promoters containing certain core promoter motifs [10,16-19]. The relative prevalence of each of these mechanisms is unknown, as in most cases is a detailed understanding of how they function. In particular, the molecular basis underlying core promoter preference has not been clearly defined.

The existence of CRM-promoter specificity is all the more remarkable given that it is maintained despite the fact that there are sometimes other promoters closer to, or even interposed between, a CRM and its target. In fact, the latter may be a much more common scenario than typically credited, as it can occur not only with respect to the regulation of different genes, but also with respect to alternative promoters of the same gene. In humans, it is estimated that upwards of 50% of all genes have at least one alternative promoter [7,20], and there is growing evidence that alternative promoter usage plays important roles in both development and disease [21]. It is unknown how frequently such alternative promoters are regulated by distinct CRMs, but the number could be large; Kimura *et al*. [20] suggest that over 1800 sets of alternative promoters are regulated in a tissue-specific fashion.

Except for the case of bidirectional promoters (those that regulate divergently transcribed genes; [22,23]), few studies have focused specifically on promoters of neighboring genes or on alternative promoters, and little is known about the mechanisms that direct promoter usage choice. Baek *et al*. [24] recently analyzed a subset of human promoters by dividing them into the four categories of CpG-island containing and non-containing single and alternative promoters, and observed differences in sequence properties, evolutionary conservation, biological roles, and degree of usage. Their data suggest that there may be differences among promoters depending on their relative position in the gene, with more upstream promoters being more highly expressed and more CpG-rich than the more downstream promoters. Interestingly, they found that the TATA box and DPE core promoter motifs were more common in single than in alternative promoters. However, a similar study by Kimura *et al*. [20] found little difference in the frequency of the TATA box between the two groups, although they observed a large difference in the prevalence of CpG islands. Differences in the full set of promoters used and in how the promoters were grouped–the latter study did not look separately at the CpG-containing and non-containing promoters–may account for the discrepancies in the reported results. A number of other sequence motifs, of unknown functional significance, were also seen to be differentially represented among the promoter classes [24]. These studies suggest that there might be fundamental differences in the structure and function of single versus alternative promoters that could have broad implications for understanding how transcription is coordinated within the genome.

As a means to developing an estimate of how important the sequence of the promoter might be in dictating promoter usage choice and in mediating CRM-promoter specificity, and as a prelude to experimental studies of the mechanisms of CRM-promoter interactions, we undertook a global bioinformatics analysis of *Drosophila melanogaster *promoters. We found that there are marked differences in nucleotide composition and motif prevalence between single promoters and alternative promoters, and between the most 5' alternative promoters and more downstream alternative promoters. We also observed that adjacent genes on the chromosome are more likely than expected to have promoters with a similar motif profile, and that this similarity in promoter configuration correlates with co-regulated gene expression. Our results suggest that promoter composition may play a larger-than-appreciated role in coordinating gene expression both between nearby genes and between multiple transcripts of the same gene.

## Results

**Figure 1:**
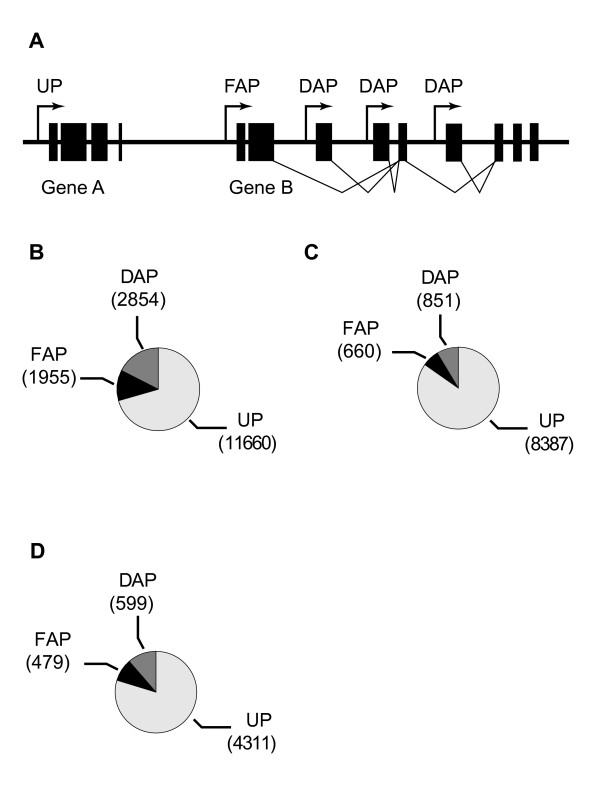
**Promoter sets used for this study**. (A) Schematic view of the three types of promoters: unique promoters (UPs), first alternative promoters (FAP), and downstream alternative promoters (DAP). (B) Number of promoters in each class when using the "all promoters" data set. (C) Number of promoters in each class when using the "high quality" data set. (D) Number of promoters in each class when using the "cap supported" data set.

In order to conduct a comprehensive genome-wide study of promoters, we looked at all protein coding genes annotated in the *Drosophila *genome annotation release 5.5 [25]. We considered all annotated TSSs to be true TSSs. Multiple TSSs that were less than 18 bp apart were considered as sharing the same promoter (see Methods). All together, we obtained 16,469 promoters from the genome, and separated these into three mutually exclusive classes (Fig. 1A; see Methods): *unique promoters *(UPs), i.e., promoters for genes having only a single promoter; *first alternative promoters *(FAPs), defined as the most 5' of a gene's multiple promoters (with respect to the coding strand); and *downstream alternative promoters *(DAPs), which are any alternative promoters 3' to the FAPs. There were 11660, 1955, and 2854 promoters in UPs, FAPs and DAPs respectively (Fig. 1B). Approximately 14% of genes had alternative promoters, with an average of 2.46 promoters/gene (range 2–13) for those with more than one promoter.

Although there are undoubtedly errors in the genome annotation with respect to TSSs [e.g., [26]], we reasoned that these errors would be few relative to the total number of annotated genes and that therefore, while they might contribute noise to our analysis, they would not mask any clearly significant results. However, to ensure the robustness of our results, we also generated two higher confidence sets of promoters: a "high quality" set of 9898 promoters based on FlyBase transcript evidence annotations and a "cap-supported" set of 5389 promoters in which all transcripts have their 5' ends accurately mapped based on cDNA isolation using a 5' cap-dependent method (see Methods). We separated these smaller promoter sets into UPs, FAPs, and DAPs, as we did with the full promoter set, and performed all analysis in parallel with the three sets (Fig. 1C, 1D). In all cases, the results were essentially the same (in a minority of cases with the two smaller-sized high-confidence datasets, values fell below the conservative statistical thresholds we had set, but trends were consistently maintained; Figure S1 [see Additional file 1], Tables S2 and S3 [see Additional file 2], and data not shown).

### Nucleotide distribution differs among the three promoter classes

**Figure 2:**
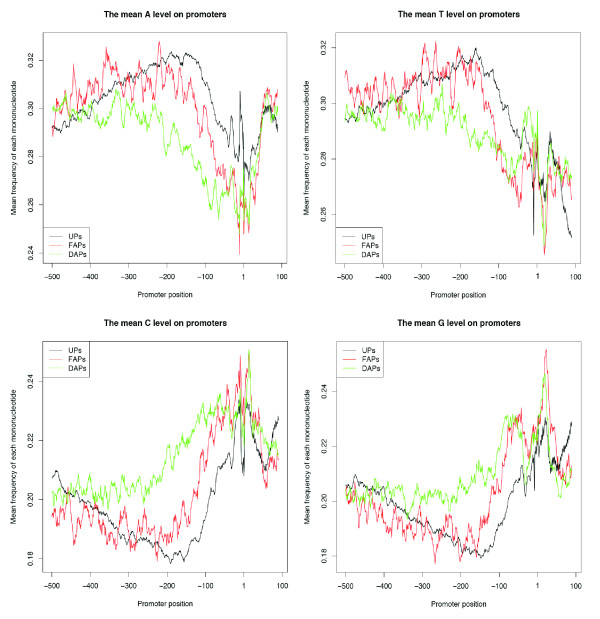
**The mononucleotide distribution of the three different classes of fly promoters**. Shown are unique promoters (black), first alternative promoters (red), the downstream alternative promoters (green). The mean frequency of each nucleotide was calculated in a 10 bp window sliding across the promoter region in 1 bp steps.

As a starting point for comparisons among the three classes of promoters, we analyzed the distribution of the four nucleotides in each class by calculating the mean frequency of each nucleotide in a 10 bp sliding window across the TSS region, ranging from -500 bp (outside the proximal and core promoter regions) to +100 bp (downstream of the core promoter). Promoters whose sequences overlapped that of an adjacent promoter of a different class were excluded from analysis. Similar to FitzGerald *et al*. [8], who looked only at UPs, we observed an AT peak around -200 bp. However, although this peak is clearly present for both UPs and FAPs, it is absent for the DAPs, which have a markedly higher GC level between -300 bp and -100 bp. Although the nucleotide distributions of UPs and FAPs are similar to one another from -500 bp to -200 bp, they begin to separate afterward: around the TSSs, UPs have more A but less C than the FAPs, and downstream of the TSS, the UPs are more GC rich than the FAPs (Fig. 2, Fig. S1). The point at which the promoter classes begin to diverge is consistent with previous findings for human genes that the region beginning at approximately -350 bp comprises an extended promoter region with an important positive regulatory role [27].

Consistent with the trends we observed in the distribution plots, we found that the GC content differs significantly among all three classes of promoters with DAP > FAP > UP (DAP versus UP: mean 0.413 (standard deviation (SD) 0.060) versus 0.379 (0.062), Kolmogorov-Smirnov test *P *≈ 0; DAP versus FAP: 0.413 (0.060) versus 0.397 (0.065), Kolmogorov-Smirnov test *P *≈ 7.46e-11; FAP versus UP: 0.397 (0.065) versus 0.379 (0.062), Kolmogorov-Smirnov test *P *≈ 4.44e-16). Note that the GC content was calculated after masking the coding regions in the promoters to avoid bias caused by the higher GC content of coding sequences [28-30]. The nucleotide distributions indicate that all three promoter classes have distinct characteristics, which are most pronounced when comparing the DAPs to the others.

### Distribution of promoter motifs in the different promoter classes

The functional units of the core promoter are the "core promoter elements," the sequences that mediate binding of the general transcription factors to the promoter. Like most transcription factor binding sites, the sequences of these elements form a family of related subsequences known as a sequence "motif." For brevity, and because in this study we do not explicitly evaluate binding but deal only with DNA sequence, we refer here to both the sequence motifs themselves and to instances of these subsequences in promoter regions as "promoter motifs." Although the relationship between various core promoter motifs and tissue- and stage-specific gene regulation has been studied previously, as have possible associations between certain motifs and genes of particular functional classes [7,8], there has been no systematic investigation of motif distributions in single versus alternative promoters, or among differently positioned alternative promoters. Therefore, we searched all 16,469 promoters for the presence of the 15 promoter motifs identified by FitzGerald *et al*. [8] in their analysis of *Drosophila *UPs that are overrepresented in core promoters or the extended promoter region up to -130 bp (Table S1 [see Additional file 2]; see Methods). A full listing of the genomic coordinates of the mapped motifs and their sequences is provided [see Additional files 3, 4, 5].

For each of the 15 motifs, we looked at their relative distributions in UPs, FAPs, and DAPs; 13 of them show a significant occurrence bias among the three promoter classes (Table 1). We see differences between unique and alternative promoters, between 5' promoters (UP or FAP) and downstream promoters, and between first alternative and more downstream alternative promoters. For example, TATA/DMp1 is found in 5.8% of UPs, 5.1-fold and 6.1-fold higher than its occurrence in the two classes of alternative promoters, FAPs and DAPs, respectively. NDM2 is significantly underrepresented in UPs as compared to the two classes of alternative promoters. DMv1, DMv2, DMv3, NDM3 and E-box/NDM5 are underrepresented in the DAPs but do not differ significantly between the UPs and FAPs. INR/DMp2 is more prevalent in DAPs than in UPs, whereas INR1/DMp3 and DPE1/DMp5 are less common in UPs than in FAPs. The presence of DMv4 and DRE/NDM4 differs significantly among all three classes of promoters with FAP > UP > DAP. GAGA/NDM1's occurrence is also significantly different among the three classes with DAP > FAP > UP. The occurrence biases we observed do not appear to correlate with the stage-specific motif usage noted by FitzGerald *et al*. [8]. For instance, TATA and GAGA are both preferentially associated with adult-expressed genes, but while TATA/DMp1 is overrepresented in UPs, GAGA/NDM1 is more prevalent in DAPs.

**Table 1:** Presence of the 15 fly promoter motifs in the three promoter classes

	Occurrence percentage^a^			p value^b^		
Motif	UPs	FAPs	DAPs	UPs vs. FAPs	UPs vs. DAPs	FAPs vs. DAPs

TATA/DMp1	5.77	1.13	0.95	9.73e-24	5.21e-36	ns

INR/DMp2	10.22	11.97	12.58	ns	3.36e-04	ns

INR1/DMp3	0.52	1.43	0.74	4.54e-05	ns	ns

DPE/DMp4	0.69	0.92	1.02	ns	ns	ns

DPE1/DMp5	0.39	1.07	0.81	3.32e-04	ns	ns

DMv1	1.56	2.46	0.70	ns	2.31e-04	6.23e-07

DMv2	0.85	0.87	0	ns	7.26e-10	2.17e-07

DMv3	3.17	3.48	1.65	ns	4.46e-06	6.86e-05

DMv4	3.39	5.58	1.23	7.10e-06	2.69e-11	5.58e-18

DMv5	1.41	0.97	0.88	ns	ns	ns

GAGA/NDM1	1.23	2.86	4.87	4.14e-07	2.68e-29	4.55e-04

NDM2	2.92	4.76	6.03	4.87e-05	3.87e-14	ns

NDM3	1.11	0.97	0	ns	8.40e-13	3.55e-08

DRE/NDM4	8.34	14.22	4.59	3.16e-15	1.04e-12	2.35e-31

E-box/NDM5	5.44	4.45	0	ns	2.73e-62	3.07e-35

None^c^	61.81	54.78	69.90	5.27e-09	4.73e-16	1.61e-26

^a^percentage of the promoters in a class having the specific motif.

^b^calculated using Fisher's exact test. Holm's method was used for multiple hypothesis testing. ns, not significant.

^c^percentage of promoters without any known motifs.

As certain combinations of promoter motifs have been shown to preferentially co-occur–for example, TATA and INR, and INR and DPE [8,31,32]–we also looked at the presence of motif combinations in the different promoter classes (see Methods). Of the 14 significantly enriched motif combinations, six show significantly different occurrence among the three promoter classes (Table 2). Overall, the pattern of motif combinations correlates with the occurrence of individual motifs. For example, DMv3, DRE/NDM4 and E-box/NDM5 are significantly underrepresented in the DAPs, and the interactions between DMv3 and DRE/NDM4 and between DRE/NDM4 and E-box/NDM5 are also underrepresented in DAPs.

**Table 2:** Presence of significant motif interactions in the three fly promoter classes

	Occurrence percentage^a^			p value^b^		
Motif interaction	UPs	FAPs	DAPs	UPs vs. FAPs	UPs vs. DAPs	FAPs vs. DAPs

TATA_INR/DMp1_DMp2	1.03	0.36	0.07	ns	1.57e-09	ns

INR_GAGA/DMp2_NDM1	0.28	0.82	0.91	1.43e-03	1.92e-05	ns

INR_NDM2/DMp2_NDM2	0.56	1.13	1.65	ns	5.84e-08	ns

DMv1_DRE/DMv1_NDM4	0.25	0.87	0.18	1.20e-04	ns	6.83e-04

DMv3_DRE/DMv3_NDM4	1.30	2.10	0.56	ns	5.66e-04	1.98e-06

DRE_E-box/NDM4_NDM5	0.97	0.77	0	ns	2.95e-11	1.33e-06

^a^percentage of the promoters in a class having the specific motif interaction.

^b^calculated using Fisher's exact test. Holm's method was used for multiple hypothesis testing. ns, not significant.

One concern in this type of analysis is that the definition of, or method of searching for, the motifs may affect the results by altering the number and locations of the identified sequences. We therefore repeated our analysis using two alternative methods for calculating the position requirement for the motifs, (Table S1 [see Additional file 2]), and also by locating motif instances using Patser [33] and the position weight matrices defined by Ohler *et al*. [34]. (While we use the weight matrices defined by Ohler, this should not be confused with use of the promoter predictions obtained in [32]. We find that there is limited correspondence between these predictions and the promoter positions obtained from the *Drosophila *annotation when requiring a prediction to fall < 18 bp from an annotated TSS–our cutoff for matching a promoter to a TSS–as opposed to the more relaxed 500 bp window allowed by Ohler; this is true even for the "high-quality" and "cap-supported" data.) All three methods gave qualitatively similar results with respect to the occurrence biases of individual motifs among the three different classes of promoters (Tables S2 and S3 [see Additional file 2]). That is, although the different methods occasionally found different absolute numbers of motif instances (e.g., Patser found more TATA boxes than regular expression methods), the motif occurrence biases among the respective promoter classes were the same.

### Human promoter classes also have distinct nucleotide compositions and motif preferences

We performed an analysis similar to that which we did for *Drosophila *using 4506 validated human promoters [24]. Nucleotide distribution plots show that both T and C levels vary among the three sets of promoters over the length of the promoter region, and A and G levels vary near the TSSs (Figure S2 [see Additional file 1]). The average GC content in human UPs is significantly higher than that in FAPs and DAPs (0.595 (0.101) versus 0.581 (0.123), Kolmogorov-Smirnov test *P *= 2.99e-06; 0.595 (0.101) versus 0.560 (0.127), Kolmogorov-Smirnov test *P *= 0). Thus while the specific nucleotide compositions of the promoters are different between flies and humans, in both species there are pronounced differences in nucleotide preference among the three promoter classes.

To determine whether human promoters also display motif preferences among the three promoter classes, as we observed in *Drosophila*, we mapped the locations of eight motifs previously identified as being overrepresented in human promoter sequences [35] (Table S4 [see Additional file 2]). Once again, we found significant differences in motif usage among the promoter classes (Table 3). In contrast to the fly promoter motifs, which collectively are distributed evenly among the promoter classes, most of the human promoter motifs have a higher occurrence frequency in UPs, and the four motifs showing occurrence bias in different classes of promoters all differ significantly between unique and alternative promoters. This compares well with the results of Baek *et al*. [24] showing a six-fold higher frequency of the TATA box in CpG-poor UPs. Our results suggest that not only does motif usage differ among human promoter classes, but that the motifs preferred in alternative promoters have not yet been identified in human. Consistent with this idea, we note that a much greater proportion of alternative promoters, as compared to unique promoters, lack any of the identified motifs we focused on in this study (53% vs. 34%).

**Table 3:** Presence of the eight human promoter motifs in the three promoter classes

	Occurrence percentage^a^			p value^b^		
Motif	UPs	FAPs	DAPs	UPs vs. FAPs	UPs vs. DAPs	FAPs vs. DAPs

CCAAT	12.25	10.16	9.78	ns	ns	ns

SP1	37.91	34.35	30.69	ns	1.33e-04	ns

CLUS1	1.82	0.81	0.50	ns	ns	ns

USF	2.76	1.94	1.61	ns	ns	ns

CREB	3.02	2.42	3.47	ns	ns	ns

TATA	6.60	1.94	3.22	6.97e-07	1.47e-04	ns

NRF-1	10.23	3.71	3.34	2.65e-08	1.89e-11	ns

ETS	17.28	5.97	6.44	1.07e-14	1.26e-16	ns

None^c^	33.59	51.77	53.59	3.75e-17	9.32e-25	ns

^a^percentage of the promoters in a class having the specific motif.

^b^calculated using Fisher's exact test. Holm's method was used for multiple hypothesis testing. ns, not significant.

^c^percentage of promoters without any known motifs.

### Promoter motif usage differs based on promoter position within a gene

The preceding analyses provide a general picture of promoter motif distributions among the different promoter classes, but do not tell us how motif profiles vary among the alternative promoters of the same gene. However, this information could provide important insights into alternative promoter use and evolution. For example, the presence of highly similar sets of motifs would suggest the possibility of extensive co-regulation of the alternative promoters. Conversely, very different motif compositions would be more consistent with differential regulation in which a distal regulatory element could interact with only one or a subset of the promoters. Landry *et al*. [36] suggest a number of ways that alternative promoters may have evolved, one of which is through local sequence duplication. This scenario would likely be reflected in higher-than-expected motif similarity, even given subsequent mutation away from the original sequence.

In order to evaluate the degree of relationship among the alternative promoters of individual genes, we compared which of the 15 promoter motifs are present between the first and second promoters of all *Drosophila *genes with alternative promoters. Each pair of promoters was then assigned one of three levels of motif similarity: *similar*, *intermediate*, or *different *(see Methods). Genes for which one or both of the two promoters contained no known motifs were excluded from the analysis.

In general, we find that the motif compositions between pairs of first and second alternative promoters are highly dissimilar. However, in comparing alternative promoter pairs, we noticed that in some genes the two promoters are close enough in position that our motif mapping rules assign the same motif to both promoters. Without experimental data, we are unable to determine whether or not such motifs, which we refer to as "putatively shared motifs," are actually used by both promoters, or just one of the pair. If we assume that putatively shared motifs are in fact used by both promoters, we find that although for the majority of the genes the first two promoters differ in motif composition, more promoter pairs are similar than we would expect at random (Table 4, "shared motifs allowed" and Figure S3 [see Additional file 6]). If we consider that a putatively shared motif is used in only one of the two promoters, however, we find that although the degree of difference in promoter motif profiles between two alternative promoters of the same gene is still different from the random expectation, the statistical support for this conclusion is weaker and not significant in all three promoter datasets (Table 4, "shared motifs not allowed" and Figure S3 [see Additional file 6]). Thus, while overall the motif composition between alternative promoters of the same gene is dissimilar, to what extent this dissimilarity is significantly less than what we would expect to see for a random pair of promoters depends on how the putatively shared motifs are actually used in the respective promoters. The answer to this question must await detailed experimental investigation.

**Table 4:** Motif similarity between the first and second promoters of the same gene^a^

	Similar^b^			Intermediate^b^			Different^b^			# of used pairs			# of omitted pairs		
	all^c^	high quality^d^	cap^e^	all	high quality	cap	all	high quality	cap	all	high quality	cap	all	high quality	cap

Observed value (shared motifs allowed)	34.07	31.52	36.04	17.70	16.30	23.42	48.23	52.17	40.54	226	92	111	1182	445	278

Observed value (shared motifs not allowed)	12.65	11.59	15.19	15.66	15.94	21.52	71.69	72.46	63.29	166	69	79	1242	468	310

Random mean^f^	9.50	8.34	8.67	10.83	12.51	14.31	79.66	79.15	77.03	226	92	111	1182	445	278

p-value^g ^(random >= observed value, shared motifs allowed)	0	0	0	2e-04	0.1399	0.0021	1	1	1	--	--	--	--	--	--

p-value^g ^(random <= observed value, shared motifs allowed)	1	1	1	1	0.9227	0.9991	0	0	0	--	--	--	--	--	--

p-value^g ^(random >= observed value, shared motifs not allowed)	0.0438	0.1286	0.0078	0.0041	0.1399	0.0108	0.9991	0.9622	1	--	--	--	--	--	--

p-value^g ^(random <= observed value, shared motifs not allowed)	0.9562	0.8714	0.9922	0.9959	0.8601	0.9892	9e-04	0.0378	0	--	--	--	--	--	--

^a^genes with exactly two alternative promoters. Similar results were obtained for genes with multiple alternative promoters (data not shown).

^b^percentage of the alternative promoter pairs defined as similar, intermediate, and different (see Methods).

^c^The promoter set contains all promoters from *Drosophila *genome annotation release 5.5.

^d^The promoter set contains only high quality promoters in fly genome; see Methods.

^e^The promoter set contains only cap-supported promoters in fly genome; see Methods.

^f^mean of 10,000 randomizations.

^g^empirical p-value calculated as the proportion of sampled randomizations where the percentage of the promoter pairs in each of the three similarity levels was no less than/no greater than the observed values

As mentioned above, promoter pairs that did not contain any of the 15 motifs in at least one promoter–approximately 84% of the potential promoter pairs (Table 4)–were omitted because we had no basis for determining how similar or different two such promoters were. Moreover, given the limited number of known promoter motifs, it is likely that for any of the promoter pairs there are additional but unidentified relevant sequence motifs. In order to get around these twin difficulties, we compared how similar the full set of promoter pairs were to one another using *D2z *scores. The *D2z *score is an alignment-free sequence comparison metric which compares *k*-mer word distributions between two sequences [37]. The alternative promoter pairs of genes with exactly two promoters were more likely to have a higher *D2z *score than random expectation (odds ratio for having *D2z *score higher than 85 percentile of random expectation = 1.44; one sided Fisher's exact *P *= 1.72e-05). Similar results were obtained for genes with multiple promoters (data not shown). This is not merely a result of the score distribution being driven by the subset of promoters with known motifs; breaking down the data into pairs for which both promoters contained known motifs, one promoter contained known motifs, or neither promoter contained known motifs revealed no differences in the score distribution for each subset (Fisher's exact *P *= 0.3594). These results indicate that for the full promoter data set, even without explicitly considering defined promoter motifs, alternative promoters of the same gene are more similar than random expectation, consistent with the results of our motif-based analysis of the smaller, motif-containing promoter subset.

### Similarities in motif composition between promoters of neighboring genes correlates with gene co-expression

Not only is the analysis of alternative promoter pairs complicated by the possibility of shared motifs, but in most cases, we do not possess good data on promoter-specific gene expression. On the other hand, questions about alternative promoter usage are paralleled by those with respect to neighboring genes: how do more distal regulatory elements select the proper promoter to activate when confronted by two or more promoters in relative proximity to one another? Unlike for alternative promoters, in the case of neighboring genes we have both unambiguous core promoter motif assignments and extensive gene expression data. Therefore, in addition to comparing promoter motif profiles among alternative promoters of the same gene, we looked at the motif composition between the promoters of neighboring genes. So as not to confound our analysis with choices as to which alternative promoters to consider, we focused on neighboring genes with single promoters only [see Additional file 7], and compared the profiles of the 15 promoter motifs in the same way that we analyzed the motif composition of alternative promoter pairs. Although the motif profiles for the majority of neighboring genes are different, we found that the number of promoters with similar motifs is significantly higher than the random expectation (Table 5 and Figure S3 [see Additional file 6]; 270/1516 vs. 129/1516, *P *≈ 0). There were a small number of neighboring unique promoters–most of which are bi-directional promoters–which, like we saw with some of the alternative promoters, could potentially share motifs. However, removing the putatively shared motifs from one or the other of the neighboring promoters did not significantly change the result (Table 5 and Figure S3 [see Additional file 6]). In other words, the promoters of neighboring genes are more closely related to one another than we would expect to see by chance alone.

**Table 5:** Motif similarity between neighboring unique promoters

	Similar^a^			Intermediate^a^			Different^a^			# of used pairs			# of omitted pairs		
	all^b^	high quality^c^	cap^d^	all	high quality	cap	all	high quality	cap	all	high quality	cap	all	high quality	cap

Observed value (shared motifs allowed)	17.81	16.44	15.47	16.36	16.90	21.13	65.83	66.67	63.40	1516	1083	530	8224	4200	983

Observed value (shared motifs not allowed)	16.64	15.06	14.34	15.50	15.91	19.96	67.86	69.03	65.70	1484	1056	516	8256	4227	997

Random mean^e^	8.48	8.25	7.60	10.45	10.96	11.96	81.07	80.79	80.44	1516	1083	530	8224	4200	983

p-value^f ^(random >= observed value, shared motifs allowed)	0	0	0	0	0	0	1	1	1	--	--	--	--	--	--

p-value^f ^(random <= observed value, shared motifs allowed)	1	1	1	1	1	1	0	0	0	--	--	--	--	--	--

p-value^f ^(random >= observed value, shared motifs not allowed)	0	0	0	0	0	0	1	1	1	--	--	--	--	--	--

p-value^f ^(random <= observed value, shared motifs not allowed)	1	1	1	1	1	1	0	0	0	--	--	--	--	--	--

^a^percentage of promoter pairs defined as similar, intermediate, and different (see Methods).

^b^The promoter set contains all promoters from *Drosophila *genome annotation release 5.5.

^c^The promoter set contains only high quality promoters in fly genome; see Methods.

^d^The promoter set contains only cap-supported promoters in fly genome; see Methods.

^e^mean of 10,000 randomizations.

^f^empirical p-value calculated as the proportion of sampled randomizations where the percentage of the promoter pairs in each of the three similarity levels was no less than/no greater than the observed values.

**Figure 3:**
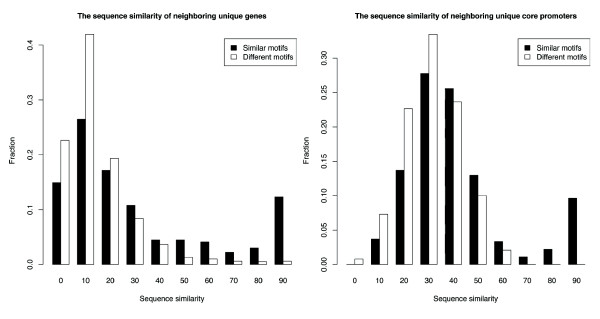
**Sequence similarity of neighboring genes and their promoters**. (A) The gene sequence similarity of neighboring unique gene pairs whose motifs are similar (black bars) or different (white bars). (B) The promoter sequence (-130 to +50 bp) similarity of neighboring unique gene pairs whose motifs are similar (black bars) or different (white bars).

One possible reason for neighboring genes to have similar promoter organizations would be if the two genes were the result of a local sequence duplication event. Indeed, we observed higher sequence similarity in both transcribed regions and in the promoter regions from -130 bp to +50 bp (in which all 15 motifs reside; Table S1 [see Additional file 2]) for neighboring unique genes that have similar motifs, compared to those with different motifs (Fig. 3). 20.5% of genes and 14.1% of promoters have greater than 65% sequence alignment for promoters with similar motif profiles, versus 2.2% and 0.5%, respectively, for promoters with different profiles (one-sided Fisher's exact *P *values = 1.52e-22 and 1.18e-21). For neighboring unique promoters with similar motifs, but not for those with disparate motifs, promoter sequence similarity is highly correlated with gene sequence similarity (*r *= 0.70, versus *r *= 0.07), indicating that not just the known motifs but the sequences of the promoter region in general are highly related. Note that the majority of genes (79%) with similar promoter motif profiles do not appear to be the result of gene duplication and that even then, promoter sequences have diverged much more rapidly than the gene sequences. Thus while potentially a contributing factor, gene duplication by itself cannot explain the unexpectedly high incidence of similar promoter motif profiles in neighboring genes.

We wondered whether the high number of neighboring genes with similar promoter make-ups might be indicative of their being coordinately regulated, e.g., by interaction of their promoters with the same enhancer. We separated the neighboring gene pairs into two groups, those for which the promoters have a similar motif profile, and those whose promoters contain a different set of motifs. We then compared the expression patterns of the genes belonging to the two different groups using gene expression data from 13 tissue-specific gene expression profiles contained in FlyAtlas [38]. For each gene pair, we calculated the degree of co-expression over the 13 tissues and found that neighboring genes whose promoters have a similar motif composition are significantly more likely to be co-expressed than neighboring gene pairs with different motifs (odds ratio of having degree of co-expression greater than 0.5 = 2.18; one sided Fisher's exact *P *= 1.04e-04).

We also calculated how well correlated the *level *of expression was for genes of both groups. For each tissue, the genes were ranked according to their mean expression level, and the Pearson correlation coefficient of the expression ranks across all 13 tissues for any two genes was obtained. Neighboring genes whose promoters have similar motif profiles were more highly correlated in their expression level than neighboring genes having different motifs (odds ratio for having a correlation coefficient in the upper quartile (>0.5) = 1.93; one sided Fisher's exact *P *= 2.97e-04).

In order to be certain that these observed correlations between gene co-expression and promoter motif composition are not an artifact resulting from their high sequence similarity, we repeated our analysis using just those neighboring gene pairs with less than 65% gene sequence similarity. In this group as well, gene pairs with similar promoter motif profiles are more likely to be co-expressed in same tissues and are more highly correlated in expression level than those with different motifs (one sided Fisher's exact *P *= 1.90e-04 and 0.002 respectively).

Our data showing that approximately 18% of neighboring genes with unique promoters have similar promoter motif compositions, and that these genes tend to have correlated expression, is consistent with previous data on co-regulation of physically near-by *Drosophila *genes [39-43]. In particular, our results are reminiscent of the finding by Spellman and Rubin [43] that roughly 20% of *Drosophila *genes fall into clusters of adjacent genes with similar expression profiles. We find that neighboring genes within one of these clusters are 63% more likely to have similar promoter motif profiles than those not within clusters (odds ratio = 1.82; one sided Fisher's exact *P *= 0.001). Thus at least some of the phenomenon described by Spellman and Rubin [43] may be attributable to similarity in core promoter motifs among the genes in a co-expression cluster.

## Discussion

### Genome organization at the promoter level

We have systematically compared the nucleotide composition and promoter motif profiles of UPs, FAPs, and DAPs throughout the *Drosophila *genome. Our results demonstrate that the three types of promoters have distinct sequence and motif preferences. Intriguingly, although consistent with results from human promoters reported by Baek *et al*. [24], we observe clear differences based not only on whether a promoter is single or alternative, but also on the relative position of an alternative promoter among all of the alternative promoters. Eight of the 15 core and proximal promoter motifs we looked at occur differentially between FAPs and DAPs, and the DAPs have a distinct nucleotide profile. Thus, the genome appears to distinguish promoter types and positions. Our results suggest that each class may be subject to different modes of regulation or interact with differently constituted basal transcription complexes, and demonstrate that the genome has a complex organizational structure at the promoter level.

It is worth noting that there is no universally accepted method for accurately identifying genomic subsequences as being relevant instances of a particular defined sequence motif, and as such, a strictly in silico analysis will always be dependent on choices of method and parameters. To guard against this, we used two different motif identification methods and three choices of range parameter for inclusion of a motif as part of the promoter. We also used three different promoter sets (four for some analyses which included the EPD set), none of which is likely to be completely accurate and which will tend to variously include too many non-promoters (false positives) or too few real promoters (false negatives). Reassuringly, we found that there were few substantive differences in our results when considering different methods and datasets; moreover, trends tended to be clearly preserved with the main differences appearing to stem from diminished statistical power when using the smaller data sets. Overall, we find our conclusions to be robust to choice of motif identification methods and search parameters, and promoter data sets.

Our analysis of a more limited number of validated human promoters revealed that just as in *Drosophila*, the different classes of promoters have distinct characteristics, and suggests that our observations point to a general metazoan organizational principle. For example, similar to what we found in the fly genome, the TATA motif is significantly overrepresented in human UPs (this study; [24]). In fact, all four significant human motifs are overrepresented in UPs, and there is a high percentage of alternative promoters that do not contain any known motifs. Thus promoter motifs in the human genome in general, and those specific to alternative promoters in particular, appear to be still awaiting identification. One reason that these motifs have proven difficult to define may lie in the abundance of alternative promoters in the human genome; the differences in motif composition and GC content among UPs, FAPs, and DAPs demonstrated by our results could contribute considerable noise to computational motif discovery attempts unless each promoter class is considered separately (see below).

### Finding additional promoter motifs

More than half of the promoters in fly genome do not contain any of the 15 motifs we used for this study (Table 1 and Figure S4 [see Additional file 8]). These promoters are problematic for purposes of promoter comparison, and some fraction of them may not in fact be true promoters, but rather might represent errors in the genome annotation that we used. However, four lines of evidence suggest that the majority of these are genuine promoters. One, we see a similar fraction of promoters lacking the known motifs when we use our more selective "high-quality" and "cap-supported" promoter sets. Two, analysis of the experimentally-verified *Drosophila *promoters in the Eukaryotic Promoter Database (EPD) [44] also reveals a high proportion without known motifs (Figure S4 [see Additional file 8]; because the EPD dataset is significantly smaller than the other three datasets we used and does not provide representative coverage of the entire genome, we did not use it for most of the analyses reported here). Three, we see similar results overall for our analysis of human promoters, which relies exclusively on experimentally-verified promoter sequences. Four, our results using the *D2z *score demonstrate that the promoters without known motifs behave identically to those that have known motifs. As a result, we believe that these represent true promoters and that a considerable number of promoter motifs remain to be identified. Indeed, a recent computational study has identified additional candidate *Drosophila *upstream promoter motifs [5], and it will be interesting to determine how these distribute with respect to the known motifs and to one another, and among the various promoter classes. Notably, all of the promoter motif discovery conducted to date has been performed on UPs [8,34] or on UPs and FAPs jointly [5]. As our data show that promoter motifs vary among the different single and alternative promoter classes, motifs specific for FAPs and DAPs may therefore be underrepresented among those that are known. Preliminary studies in our laboratory suggest that targeting motif discovery efforts to specific promoter subsets will be an effective strategy for identifying new motifs (J. Spix, QZ, and MSH, unpublished results).

### Promoters and gene expression neighborhoods

The majority of adjacent promoters, either from neighboring genes or from alternative promoters of the same gene, have a highly dissimilar motif profile. As promoter motifs have been implicated in helping to mediate specific enhancer-promoter interactions [10,16-19], these differences are likely to represent one of the mechanisms used by the genome to prevent inappropriate gene activation by nearby CRMs. Nevertheless, neighboring genes are significantly more likely than expected to have highly similar promoters, with a strong correlation between motif similarity and strength of gene expression. Thus, a key role of the promoter may be in regulating levels of gene expression. Neighboring genes with similar promoters also show a concomitant increase in tissue co-expression, raising the possibility that they are either coordinately regulated by shared CRMs [e.g. [45]], or by individual CRMs that bind a similar complement of transcription factors [46]. Neighborhoods of co-regulated genes have been observed in many eukaryotes, including yeast, worm, fly, and human [39-43,45-51]. A frequently proposed mechanism for this phenomenon is the presence of local chromatin domains; that is, that a local "open" chromatin configuration favorable for transcription–perhaps due to strong activation of one of the genes in the neighborhood–leads to spurious activation of other nearby genes, as opposed to regulated activation of each gene [43]. In contrast, our data suggest that promoter sequences constitute a significant component of gene co-expression neighborhoods and point to a higher degree of genomic organization and regulation than seen for a chromatin-centric model. Note that the two mechanisms need not be mutually exclusive; for instance, the related promoters might all contribute to a strong local change in chromatin conformation that would affect even those neighboring genes with different promoter make-ups. Detailed experimental investigation along with careful mapping of chromatin modifications throughout the co-expression neighborhoods will be required to tease apart the various contributions of individual regulatory *cis*-*trans *interactions and epigenetic modifications. However, our results suggest a much greater role for the promoter in mediating locally coordinated gene expression than heretofore appreciated.

## Conclusions

Our systematic investigation of *Drosophila *promoters demonstrates that there are distinct sequence characteristics among unique, first alternative, and downstream alternative promoters, suggesting that different regulatory mechanisms may act preferentially on each class. We also show that neighboring genes are unexpectedly likely to have similar promoter compositions, which correlates with an increased degree of gene coexpression and suggests a mechanism for the previously observed phenomenon of gene co-expression neighborhoods. Taken together, these data reveal a high degree of complex genome organization at the level of promoter sequences.

## Methods

### Promoter datasets

*Drosophila *release 5.5 genomic sequences and annotation were downloaded from FlyBase [25]. The given start position of each mRNA which has strand information was considered as the position of the TSS, and the sequences from -500 bp to +100 bp were extracted as the extended promoter regions. The upstream region of one TSS was shorter than 500 bp and therefore not included in the dataset. We considered transcripts within 18 bp of one another to correspond to the same promoter based on an analysis of the spacing between multiple TSSs of individual genes (Figure S5A [see Additional file 9]). In such cases, the mean position of the multiple TSSs was used to define a representative TSS. Then the extended promoter region was defined as -500 bp to +100 bp around the representative TSS. Promoters were separated into three different groups based on their relative positions in the gene. As no significant differences were observed between downstream alternative promoters (DAPs) irrespective of their order following the most 5' promoter (data not shown), we grouped these promoters together into a single class in order to take advantage of the increased statistical power of the larger sample size. Genes whose promoters could not be assigned unambiguously to one of the three groups were removed.

To obtain the "high quality" *Drosophila *promoter set, we used the transcript evidence rank provided by FlyBase in annotation release 5.5. We considered a transcript, and thus its TSS, to be strongly supported if it had a FlyBase evidence score of nine or more. In order to achieve such a score, a transcript must have one or more aligned cDNA sequences that are fully consistent with the annotation, plus at least one of the following: one or more consistent aligned EST sequences; intersection of an annotated exon with a region of aligned protein similarity; or a gene prediction fully consistent with the annotation. We extracted the promoters only from these strongly supported transcripts. To make sure the separation of promoters into UPs, FAPs and DAPs was reliable, we also required that at least the first two transcripts of a gene were strongly supported.

To obtain the cap-supported *Drosophila *promoters, we used cDNA and 5' EST sequences from four 5' cap-trapped cDNA libraries (RE, RH, TA, and TB) [52,53]. The genomic coordinates of the 5' end of these cDNA and 5' ESTs were extracted from the fly genome annotation by requiring the first 5 bp of the 5' end to map to the genome. We considered a transcript, and thus its TSS, to be cap-supported if the TSS is within 10 bp of the 5' end coordinates of a cap-trapped cDNA or EST. To make sure the separation of promoters into UPs, FAPs and DAPs was reliable, we also required that at least the first two transcripts of a gene were strongly supported. Transcripts within 17 bp of one another were considered as corresponding to the same promoter based on an analysis of the spacing between multiple cap-supported TSSs of individual genes (Figure S5B [see Additional file 9]).

The coordinates of the single and alternative human promoters identified by Baek *et al*. [24] were obtained by mapping the corresponding transcripts to EST, mRNA and RefGene of human genome annotation (hg17) in UCSC Genome Browser [54]. We grouped transcripts that belonged to the same gene according to Baek *et al*. to get all transcripts of one gene. We removed transcripts that were inconsistent between Baek *et al*. [24] and current human annotation. Sequences from -500 bp to +100 bp of the first nucleotide of the relevant transcripts were defined as the promoter regions. A total of 4506 human promoters were obtained.

### Mononucleotide distribution and GC content

The program *freak *from the EMBOSS software suite [55] was used to calculate the frequency of each nucleotide in a 10 bp window moving along the promoter sequence in 1 bp steps. GC content was calculated using the EMBOSS *geecee *program. Coding regions were masked so that only non-coding sequences were considered. For both analyses, any promoters whose sequences overlapped were excluded for a total n = 11633 UPs, 1058 FAPs, and 1850 DAPs for *Drosophila *promoters and n = 3078 UPs, 509 FAPs, and 696 DAPs for human promoters.

### Promoter Motifs

The consensus sequences and strand specificities of the 15 *Drosophila *promoter motifs are given by FitzGerald *et al*. [8]. Both strands of the promoter sequence were searched for the degenerate consensus sequences using the EMBOSS program *fuzznuc *allowing zero mismatches. We computed the distribution of each motif relative to the TSS in 20 bp bins for promoters using R [56]. The valid range of each motif was taken to be the bins whose frequencies were above two standard deviations of the mean frequency of all 30 bins along the promoter regions [8,35]. We calculated the valid ranges ("subset range") of every motif on each of the three types of promoters (UPs/FAPs/DAPs). When no valid range for a motif in a promoter class could be detected (i.e., frequency was equal to background), the motif was considered to be absent from the promoter class and the valid range was set to zero. In the case where two adjacent bins, or two bins with a single intervening bin satisfied the above criteria, the entire region was used as the valid range. Because the numbers of FAPs and DAPs was relatively small for some of the promoter data sets, ranges were also calculated by considering all three classes of promoters together, for purpose of comparison ("combined range"). We also used position requirements for the motifs from FitzGerald *et al*. [8] ("literature-based"). For each analysis, hits that matched the consensus sequence but which were on the wrong strand, or which fell outside of the valid range, were considered false positive hits and were excluded from analysis.

For weight-matrix based motif searching, position-specific scoring matrices (PSSMs) corresponding to the ten motifs identified by Ohler *et al*. [34] were used to scan the promoter sequences with Patser [33]. Motif ten was removed from further analysis because it did not match to any of the 15 motifs identified by FitzGerald *et al*. [8] (Table S1 [see Additional file 2]). We imposed strand and position requirements as described above for determining whether to accept identified motifs as true hits. Cutoff values for Patser were chosen as follows. We first converted the PSSMs from Ohler *et al*. [34] to position-specific probability matrices (PSPMs). We also generated a PSPM from each set of sequences found by Patser at each cutoff value from e-03 to e-12. The PSPMs for each motif were clustered based on Euclidian distance using the PAM function in R [56] and the clustering result with the maximum average silhouette was chosen. The highest Patser P-value that fell within the same cluster as the original PSPM from Ohler *et al*. [34] was used as the cutoff for that specific motif (Table S1 [see Additional file 2]).

The eight human promoter motifs were originally identified by FitzGerald *et al*. [35]. Strand and range information for these motifs were taken directly from the literature. The consensus sequences of these eight motifs were taken from Fig. 9 of FitzGerald *et al*. [8].

### Promoter motif interactions

For every possible combination of two fly promoter motifs, we counted the number of all promoters in the genome that contain both motifs, the number of promoters containing only one of the two motifs, and the number of promoters containing neither of the two motifs to make a 2 × 2 contingency table. Fisher's exact test was then used on each of the contingency tables to test the association between the two corresponding motifs. 14 of a total 105 motif combinations showed significant positive associations after correcting for multiple hypothesis tests using Holm's method.

### Motif profile similarity

Motifs in all promoters were compiled into a 16469 × 15 matrix in which each row represented a promoter and each column, a motif. Motif presence was indicated by "one," absence by "zero," so that the motif occurrence matrix contained only binary values. We calculated the distance in the promoter motifs between any two promoters by using the *dist *function in R [56] with the asymmetric binary distance measure. The distance between any two promoters is therefore defined as the number of motifs which only occur in one of the two promoters divided by the total number of motifs that occur in at least one of the two promoters. When the distance was less than 0.2, the motifs in the two promoters were called *similar; *distances of greater than or equal to 0.8 were defined as *different*. Promoter pairs in which either of the promoters did not contain one of the 15 identified motifs were omitted from the analysis.

When two alternative promoters are very close in position, our motif mapping rules could sometimes assign the same motif to both promoters. To obtain results for which such shared motifs were not allowed, we ignored the occurrence of the shared motifs on the first of a pair alternative promoters and compared the motif profiles as described above. Similar results were obtained when ignoring the shared motifs on the second promoter of each pair (data not shown).

To calculate random expectations, we broke the pairing between alternative promoters of the same gene, or neighboring unique promoters, and randomized the pairing 10,000 times for each type.

### D2z score

*D2z *scores were calculated using a promoter region of -300 bp to +100 bp from TSSs, as *D2z *score has standard normal distribution when the sequence length is at least 400 bp [37]. The *D2z *score between promoter sequences was calculated using word length five and background Markov Model order zero, the parameter setting which performed best in distinguishing functionally related regulatory sequences from not-related sequences [37]. If the sequences of an alternative promoter pair belonging to the same gene overlapped in position, the pair was removed from analysis. The *D2z *scores of the incorrectly paired promoter sequences were used as random expectation.

### Gene expression data

Gene expression data for 13 tissues were obtained from FlyAtlas [38]. We considered a gene to be expressed in a tissue if it was called present in greater than 50% of the replicate microarrays reported in FlyAtlas. The tissue expression similarity of two genes was defined as the fraction of the 13 tissues in which the genes are either both present or both absent.

To calculate expression level correlations, we first ranked all genes according to their mean expression level in each of the 13 tissues using the *rank *function in R (with the rank of ties equal to average rank) and then computed the Pearson correlation coefficient of the ranks for the two genes being compared.

### Sequence similarity

Sequence similarity was assessed using Dialign [57] and reported as the maximum fraction of aligned residues between the two sequences.

## Abbreviations

TSS: transcription start site; TBP: TATA box-binding protein; TRFs: TBP-related factors; CRM: *cis*-regulatory module; UPs: unique promoters; FAPs: first alternative promoters; DAPs: downstream alternative promoters; EPD: the Eukaryotic Promoter Database; PSSM: Position-specific scoring matrix; PSPM: position-specific probability matrix.

## Authors' contributions

Both authors conceived and designed the study and wrote the manuscript. QZ performed the analysis.

## Supplementary Material

Additional file 1 **Figures S1 and S2**. Mononucleotide distributions of the three groups of promoters when using "high quality" fly promoters (S1A), cap-supported fly promoters (S1B) and human promoter sets (S2). Click here for file

Additional file 2 **Tables S1-S4. **Table S1: *Drosophila *promoter motifs used in this study; Table S2: Motif preferences when using different motif position requirements; Table S3: Motif preferences when using regular expressions versus position weight matrices to represent motifs; Table S4: Human promoter motifs used in this study. Click here for file

Additional file 3 **Promoter motif coordinates from our primary study**. This file contains all of the mapped motifs from our primary study formatted for upload to the Generic Genome Browser (GBrowse) as a custom annotation. Click here for file

Additional file 4 **Promoter motif coordinates using "combined range"**. This file contains all of the mapped motifs using the motif definitions and position requirements "combined range" formatted for upload to the Generic Genome Browser (GBrowse) as a custom annotation. Click here for file

Additional file 5 **Promoter motif coordinates from Patser**. This file contains all of the mapped motifs using Patser, as described in the text, formatted for upload to the Generic Genome Browser (GBrowse) as a custom annotation. Click here for file

Additional file 6 **Figure S3**. Motif dissimilarity distributions between alternative promoters of the same gene and between neighboring unique promoters. Click here for file

Additional file 7 **neighboring single-promoter genes**. This file contains the names and coordinates of the pairs of neighboring single-promoter genes used for the analysis presented in Table 5 and Figure 3. Click here for file

Additional file 8 **Figure S4**. Distribution of the number of mapped motifs in individual promoters. Click here for file

Additional file 9 **Figure S5**. Histogram of distances between alternative TSSs for the same gene in the fly genome when all promoters in the genome (A) or only cap-supported promoters (B) were considered. Click here for file
