# Supplementary material for: Complex organizational structure of the genome revealed by genome-wide analysis of single and alternative promoters in Drosophila melanogaster
Source: BMC Genomics. 2009 Jan 7;10:9. doi: 10.1186/1471-2164-10-9 (PMC2631479; doi:10.1186/1471-2164-10-9)
Supplement: Additional file 1 — Figures S1 and S2. Mononucleotide distributions of the three groups of promoters when using "high quality" fly promoters (S1A), cap-supported fly promoters (S1B) and human promoter sets (S2). [file 1471-2164-10-9-S1.pdf]

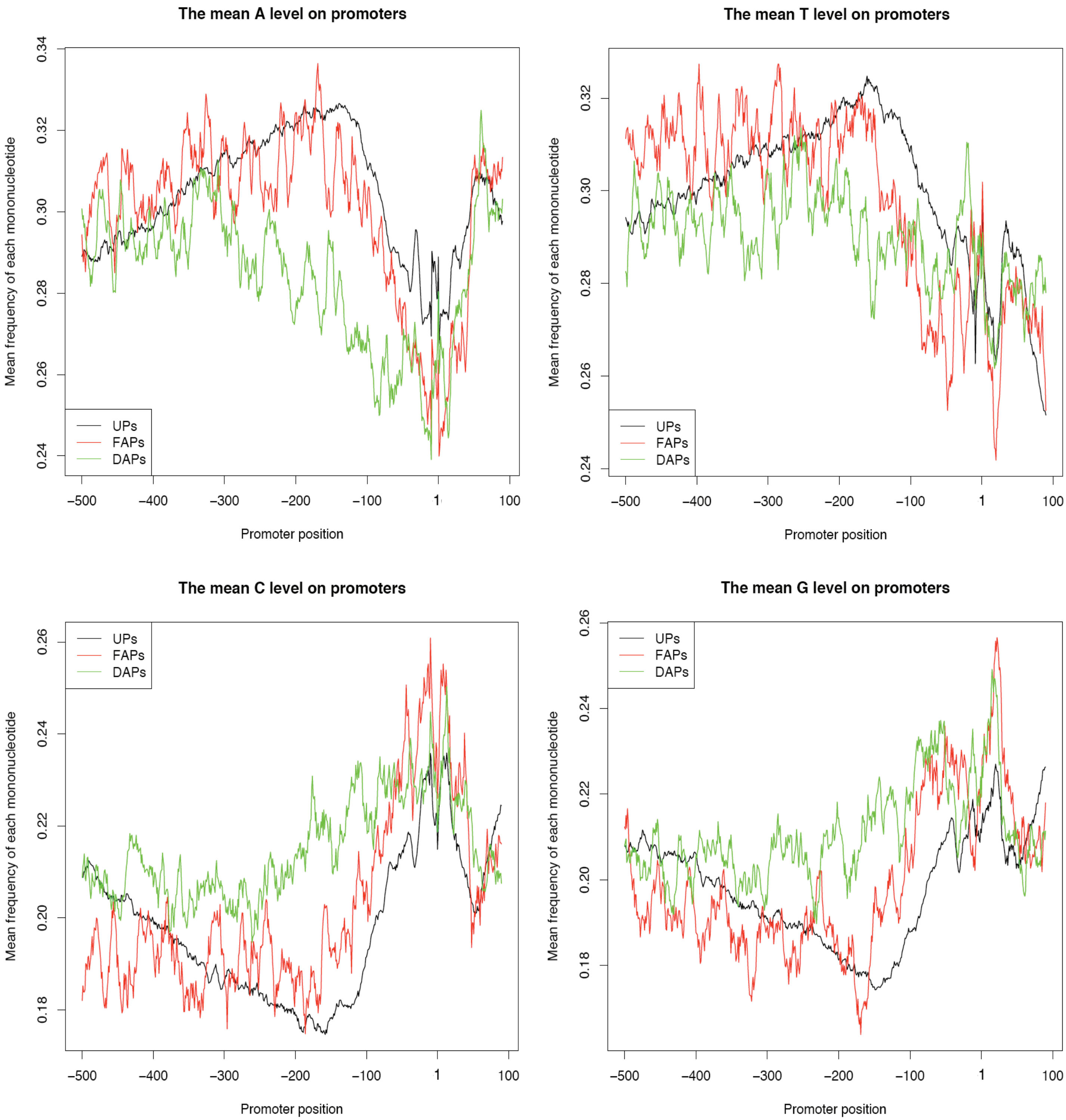

Figure S1A: Mononucleotide distributions of the three groups of promoters when using “high quality” fly promoters. Shown are unique promoters (black), first alternative promoters (red), the downstream alternative promoters (green). The mean frequency of each nucleotide was calculated in a 10 bp window sliding across the promoter region in 1 bp steps.

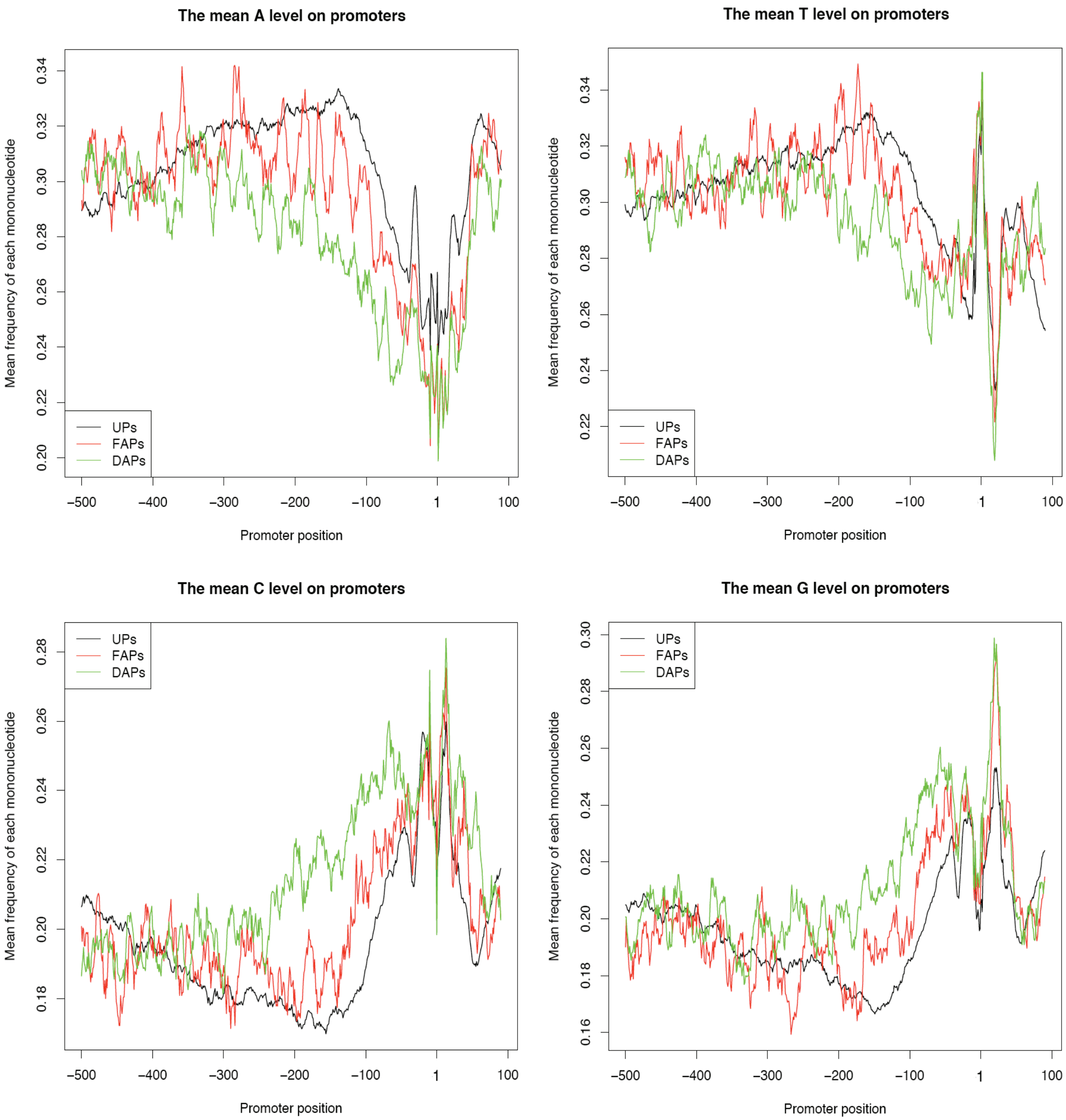

Figure S1B: Mononucleotide distributions of the three groups of promoters when using cap-supported fly promoters. Shown are unique promoters (black), first alternative promoters (red), the downstream alternative promoters (green). The mean frequency of each nucleotide was calculated in a 10 bp window sliding across the promoter region in 1 bp steps.

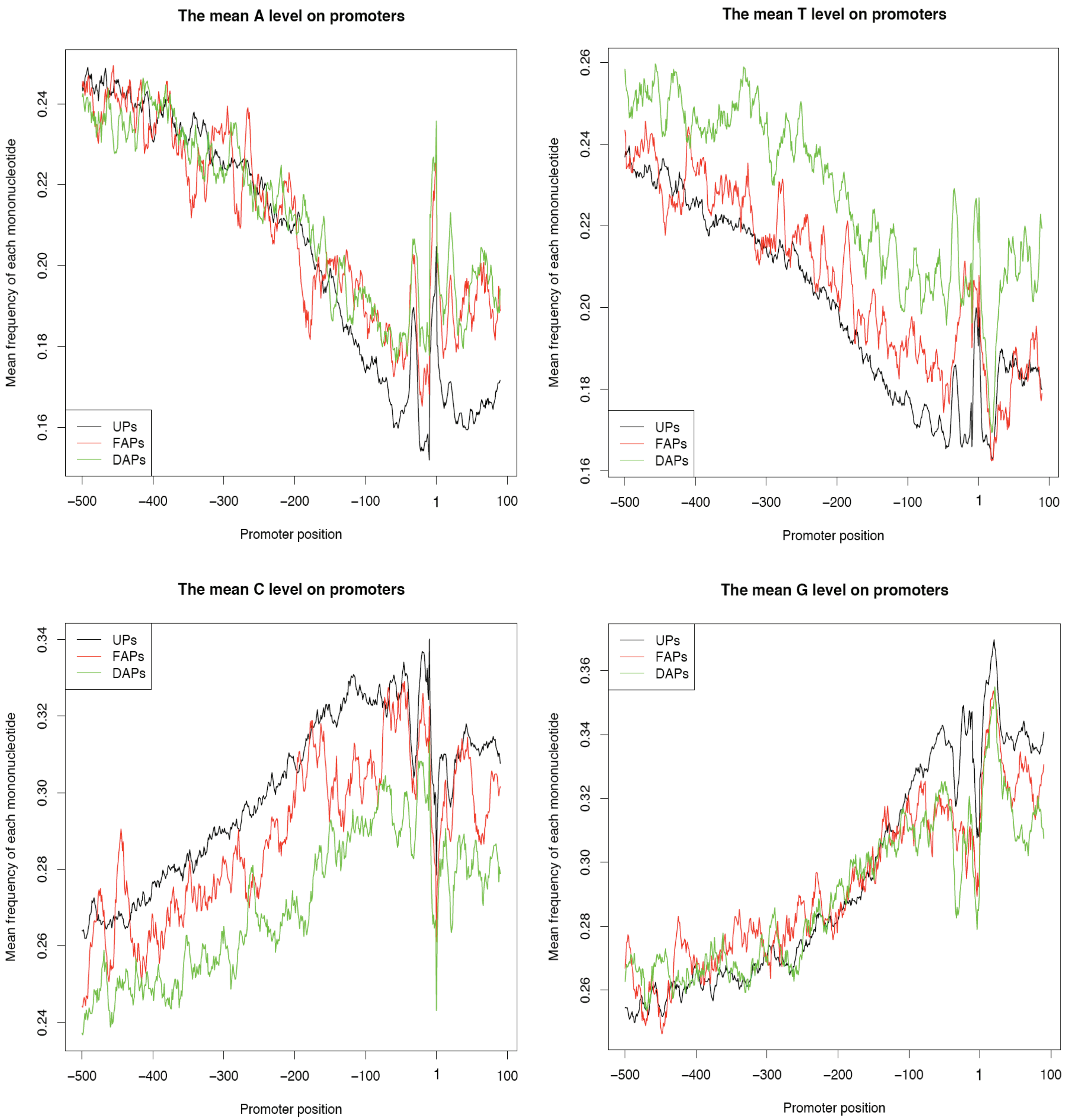

Figure S2: The mononucleotide distribution of the three different groups of human promoters. Shown are unique promoters (black), first alternative promoters (red), the downstream alternative promoters (green). The mean frequency of each nucleotide was calculated in a 10 bp window sliding across the promoter region in 1 bp steps.
