# Supplementary material for: Complex organizational structure of the genome revealed by genome-wide analysis of single and alternative promoters in Drosophila melanogaster
Source: BMC Genomics. 2009 Jan 7;10:9. doi: 10.1186/1471-2164-10-9 (PMC2631479; doi:10.1186/1471-2164-10-9)
Supplement: Additional file 2 — Tables S1-S4. Table S1: Drosophila promoter motifs used in this study; Table S2: Motif preferences when using different motif position requirements; Table S3: Motif preferences when using regular expressions versus position weight matrices to represent motifs; Table S4: Human promoter motifs used in this study. [file 1471-2164-10-9-S2.pdf]

**Additional file 2: Zhu and Halfon**

Table S1: The 15 fly promoter motifs[a]

| Motif | Consensus sequence[b] | Common name | Strand[c] | valid range (subset) [d] | | | valid range (combined)[e] | valid range (literature-based)[f] | Ohler #[g] | Patser cutoff[h] | Patser cutoff[i] |
|---|---|---|---|---|---|---|---|---|---|---|---|
| | | | | UPs | FAPs | DAPs | | | | | |
| DMp1 | STATAAA | TATA | + | -60 to -21 | -40 to -21 | -40 to -21 | -60 to -21 | -60 to -21 | 3 | e-8 | e-8 |
| DMp2 | TCAGTY | INR | + | -20 to 20 | -20 to 20 | -20 to 20 | -20 to 20 | -40 to 20 | 4 | e-7 | e-7 |
| DMp3 | TCATTCG | INR1 | + | 1 to 20 | -20 to 20 | 1 to 20 | -20 to 20 | -20 to 20 | | | |
| DMp4 | KCGGTTSK | DPE | + | 21 to 40 | 21 to 40 | 21 to 40 | 21 to 40 | 1 to 40 | 9 | e-8 | e-8 |
| DMp5 | CGGACGT | DPE1 | + | 21 to 40 | 1 to 40 | 1 to 40 | 21 to 40 | 1 to 40 | | | |
| DMv1 | CARCCCT | | + | -60 to -1 | -40 to 20 | -60 to -41 | -60 to -1 | -80 to 20 | | | |
| DMv2 | TGGYAACR | | + | -20 to -1 | -40 to -21 | 0 | -20 to -1 | -100 to 20 | 8 | e-9 | e-9 |
| DMv3 | CAYCNCTA | | + | -40 to 20 | -20 to 20 | -60 to -1 | -20 to 20 | -100 to 40 | 7 | e-9 | e-8 |
| DMv4 | GGYCACAC | | + | -20 to 20 | -20 to 40 | -40 to -1 | -20 to 20 | -100 to 20 | 1 | e-8 | e-8 |
| DMv5 | TGGTATTT | | + | -60 to -1 | -40 to -21 | -100 to -41 | -60 to -1 | -120 to 20 | 6 | e-8 | e-8 |
| NDM1 | GAGAGCG | GAGA | both | -100 to -61 | -100 to -61 | -120 to -61 | -120 to -61 | -140 to -61 | | | |
| NDM2 | CGMYGYCR | | both | -80 to -41 | -80 to -41 | -100 to -41 | -80 to -41 | -120 to -61 | | | |
| NDM3 | GAAAGCT | | both | -80 to -41 | -80 to -61 | 0 | -80 to -41 | -140 to -61 | | | |
| NDM4 | ATCGATA | DRE | both | -60 to -1 | -80 to -1 | -100 to -41 | -60 to -21 | -120 to 20 | 2 | e-8 | e-8 |
| NDM5 | CAGCTSWW | E-box | both | -40 to 20 | -60 to -1 | 0 | -40 to 20 | -100 to 40 | 5 | e-8 | e-8 |

[a]As given by FitzGerald *et al*. (2006)

[b]R=G, A; W=A, T; Y=T, C; K=G, T; M=A, C; S=G, C; N=A, T, G, C

[c]+, coding strand; both, either strand

[d]relative to the TSS; range was calculated for each of the three types of promoters; see Methods

[e]relative to the TSS; range was calculated after combining the three types of promoters; see Methods

[f]relative to the TSS, as given by FitzGerald *et al*. (2006)

[g]the correspondence between the 15 motifs and the motifs found by Ohler *et al*. (2002) as given in Figure 5 of FitzGerald *et al*. (2006)

[h]the Patser cutoff used to search the motifs found by Ohler *et al*. (2002) in all fly promoters; see Methods

[i]the Patser cutoff used to search the motifs found by Ohler *et al*. (2002) in high quality fly promoters; the calculated Patser cutoffs for cap-supported promoters are the same as the cutoffs calculated for high quality promoters; see Methods

# Table S2: The $log_2$ relative probability of fly promoter motif present in any two of the three promoter classes

| Motif | | UPs vs. FAPs | | | UPs vs. DAPs | | | FAPs vs. DAPs | | |
|---|---|---|---|---|---|---|---|---|---|---|
| | | Subset range[a] | Combined range[b] | Literature-based[c] | Subset range | Combined range | Literature-based | Subset range | Combined range | Literature-based |
| TATA/DMp1 | all[d] | **+2.36 (5.77, 1.13)**[h] | **+1.46 (5.77, 2.1)** | **+1.46 (5.77, 2.1)** | **+2.61 (5.77, 0.95)** | **+2.08 (5.77, 1.37)** | **+2.08 (5.77, 1.37)** | +0.25 (1.13, 0.95) | +0.62 (2.1, 1.37) | +0.62 (2.1, 1.37) |
| TATA/DMp1 | high_quality[e] | **+2.22 (5.65, 1.21)** | **+1.05 (5.65, 2.73)** | **+1.05 (5.65, 2.73)** | **+2.27 (5.65, 1.18)** | **+1.78 (5.65, 1.65)** | **+1.78 (5.65, 1.65)** | +0.04 (1.21, 1.18) | +0.73 (2.73, 1.65) | +0.73 (2.73, 1.65) |
| TATA/DMp1 | cap[f] | **+3.12 (7.28, 0.84)** | **+2.8 (7.28, 1.04)** | **+2.8 (7.28, 1.04)** | **+2.13 (7.28, 1.67)** | **+2.13 (7.28, 1.67)** | **+2.13 (7.28, 1.67)** | -1 (0.84, 1.67) | -0.68 (1.04, 1.67) | -0.68 (1.04, 1.67) |
| TATA/DMp1 | EPD[g] | +1.98 (7, 1.77) | +1.98 (7, 1.77) | +1.98 (7, 1.77) | +1.11 (7, 3.25) | +1.11 (7, 3.25) | +1.11 (7, 3.25) | -0.88 (1.77, 3.25) | -0.88 (1.77, 3.25) | -0.88 (1.77, 3.25) |
| INR/DMp2 | all | -0.23 (10.22, 11.97) | -0.23 (10.22, 11.97) | -0.08 (12.8, 13.5) | **-0.3 (10.22, 12.58)** | **-0.3 (10.22, 12.58)** | **-0.29 (12.8, 15.59)** | -0.07 (11.97, 12.58) | -0.07 (11.97, 12.58) | -0.21 (13.5, 15.59) |
| INR/DMp2 | high_quality | +0.19 (11.42, 10) | +0.19 (11.42, 10) | +0.18 (13.41, 11.82) | -0.17 (11.42, 12.81) | -0.17 (11.42, 12.81) | -0.19 (13.41, 15.28) | -0.36 (10, 12.81) | -0.36 (10, 12.81) | -0.37 (11.82, 15.28) |
| INR/DMp2 | cap | -0.01 (14.71, 14.82) | -0.01 (14.71, 14.82) | -0.05 (15.36, 15.87) | -0.28 (14.71, 17.86) | -0.28 (14.71, 17.86) | -0.31 (15.36, 19.03) | -0.27 (14.82, 17.86) | -0.27 (14.82, 17.86) | -0.26 (15.87, 19.03) |
| INR/DMp2 | EPD | -0.28 (14.54, 17.7) | -0.28 (14.54, 17.7) | -0.21 (15.25, 17.7) | **-1.09 (14.54, 30.89)** | **-1.09 (14.54, 30.89)** | **-1.02 (15.25, 30.89)** | -0.8 (17.7, 30.89) | -0.8 (17.7, 30.89) | -0.8 (17.7, 30.89) |
| INR1/DMp3 | all | **-1.45 (0.52, 1.43)** | -0.83 (0.81, 1.43) | -0.83 (0.81, 1.43) | -0.49 (0.52, 0.74) | -0.48 (0.81, 1.12) | -0.48 (0.81, 1.12) | +0.96 (1.43, 0.74) | +0.35 (1.43, 1.12) | +0.35 (1.43, 1.12) |
| INR1/DMp3 | high_quality | -1.4 (0.63, 1.67) | -0.9 (0.89, 1.67) | -0.9 (0.89, 1.67) | -0.38 (0.63, 0.82) | -0.39 (0.89, 1.18) | -0.39 (0.89, 1.18) | +1.02 (1.67, 0.82) | +0.5 (1.67, 1.18) | +0.5 (1.67, 1.18) |
| INR1/DMp3 | cap | +0.09 (1.11, 1.04) | +0.18 (1.18, 1.04) | +0.18 (1.18, 1.04) | -0.58 (1.11, 1.67) | -0.63 (1.18, 1.84) | -0.63 (1.18, 1.84) | -0.68 (1.04, 1.67) | -0.81 (1.04, 1.84) | -0.81 (1.04, 1.84) |
| INR1/DMp3 | EPD | -1.9 (1.19, 4.42) | -1.18 (1.96, 4.42) | -1.18 (1.96, 4.42) | -2.04 (1.19, 4.88) | -1.54 (1.96, 5.69) | -1.54 (1.96, 5.69) | -0.14 (4.42, 4.88) | -0.36 (4.42, 5.69) | -0.36 (4.42, 5.69) |
| DPE/DMp4 | all | -0.41 (0.69, 0.92) | -0.41 (0.69, 0.92) | -0.36 (1.04, 1.33) | -0.55 (0.69, 1.02) | -0.55 (0.69, 1.02) | -0.6 (1.04, 1.58) | -0.14 (0.92, 1.02) | -0.14 (0.92, 1.02) | -0.25 (1.33, 1.58) |
| DPE/DMp4 | high_quality | +0.12 (0.82, 0.76) | +0.12 (0.82, 0.76) | -0.17 (1.22, 1.36) | -0.19 (0.82, 0.94) | -0.19 (0.82, 0.94) | +0.05 (1.22, 1.18) | -0.31 (0.76, 0.94) | -0.31 (0.76, 0.94) | +0.21 (1.36, 1.18) |
| DPE/DMp4 | cap | -0.02 (1.44, 1.46) | -0.02 (1.44, 1.46) | +0.23 (1.72, 1.46) | -0.06 (1.44, 1.5) | -0.06 (1.44, 1.5) | +0.04 (1.72, 1.67) | -0.04 (1.46, 1.5) | -0.04 (1.46, 1.5) | -0.19 (1.46, 1.67) |
| DPE/DMp4 | EPD | +0.69 (1.42, 0.88) | +0.69 (1.42, 0.88) | +1.27 (2.14, 0.88) | -0.19 (1.42, 1.63) | -0.19 (1.42, 1.63) | -0.19 (2.14, 2.44) | -0.88 (0.88, 1.63) | -0.88 (0.88, 1.63) | -1.46 (0.88, 2.44) |
| DPE1/DMp5 | all | **-1.45 (0.39, 1.07)** | -0.86 (0.39, 0.72) | -0.9 (0.57, 1.07) | -1.03 (0.39, 0.81) | -0.41 (0.39, 0.53) | -0.49 (0.57, 0.81) | +0.41 (1.07, 0.81) | +0.45 (0.72, 0.53) | +0.41 (1.07, 0.81) |
| DPE1/DMp5 | high_quality | -1.35 (0.48, 1.21) | -0.93 (0.48, 0.91) | -0.83 (0.68, 1.21) | -1.44 (0.48, 1.29) | -0.79 (0.48, 0.82) | -0.93 (0.68, 1.29) | -0.09 (1.21, 1.29) | +0.14 (0.91, 0.82) | -0.09 (1.21, 1.29) |
| DPE1/DMp5 | cap | +0.12 (0.9, 0.84) | +0.12 (0.9, 0.84) | +0.26 (1, 0.84) | +0.12 (0.9, 0.83) | +0.12 (0.9, 0.83) | +0.26 (1, 0.83) | 0 (0.84, 0.83) | 0 (0.84, 0.83) | 0 (0.84, 0.83) |
| DPE1/DMp5 | EPD | -0.73 (1.07, 1.77) | -0.73 (1.07, 1.77) | -0.73 (1.07, 1.77) | -0.61 (1.07, 1.63) | +0.39 (1.07, 0.81) | -0.61 (1.07, 1.63) | +0.12 (1.77, 1.63) | +1.12 (1.77, 0.81) | +0.12 (1.77, 1.63) |
| DMv1 | all | -0.65 (1.56, 2.46) | -0.62 (1.56, 2.4) | -0.55 (2.3, 3.38) | **+1.16 (1.56, 0.7)** | +0.19 (1.56, 1.37) | +0.18 (2.3, 2.03) | **+1.81 (2.46, 0.7)** | +0.82 (2.4, 1.37) | +0.73 (3.38, 2.03) |
| DMv1 | high_quality | -0.38 (1.86, 2.42) | -0.19 (1.86, 2.12) | -0.26 (2.79, 3.33) | +1.66 (1.86, 0.59) | +0.66 (1.86, 1.18) | +0.32 (2.79, 2.23) | +2.04 (2.42, 0.59) | +0.85 (2.12, 1.18) | +0.58 (3.33, 2.23) |
| DMv1 | cap | -0.18 (2.57, 2.92) | -0.38 (2.57, 3.34) | -0.03 (3.9, 3.97) | +0.78 (2.57, 1.5) | +0.36 (2.57, 2) | +0.54 (3.9, 2.67) | +0.96 (2.92, 1.5) | +0.74 (3.34, 2) | +0.57 (3.97, 2.67) |
| DMv1 | EPD | +1.17 (3.98, 1.77) | +1.17 (3.98, 1.77) | +0.24 (5.22, 4.42) | +2.29 (3.98, 0.81) | +2.29 (3.98, 0.81) | +2.68 (5.22, 0.81) | +1.12 (1.77, 0.81) | +1.12 (1.77, 0.81) | +2.44 (4.42, 0.81) |
| DMv2 | all | -0.03 (0.85, 0.87) | +0.59 (0.85, 0.56) | -0.04 (2.74, 2.81) | **+Inf (0.85, 0)** | +1.43 (0.85, 0.32) | +0.59 (2.74, 1.82) | **+Inf (0.87, 0)** | +0.84 (0.56, 0.32) | +0.63 (2.81, 1.82) |
| DMv2 | high_quality | +0.07 (0.95, 0.91) | +1.65 (0.95, 0.3) | +0.28 (3.14, 2.58) | **+Inf (0.95, 0)** | +1.44 (0.95, 0.35) | +0.93 (3.14, 1.65) | +Inf (0.91, 0) | -0.22 (0.3, 0.35) | +0.65 (2.58, 1.65) |
| DMv2 | cap | +1.44 (1.14, 0.42) | +0.44 (1.14, 0.84) | +0.22 (3.64, 3.13) | +Inf (1.14, 0) | +Inf (1.14, 0) | **+1.86 (3.64, 1)** | +Inf (0.42, 0) | +Inf (0.84, 0) | +1.64 (3.13, 1) |
| DMv2 | EPD | +0.01 (0.89, 0.88) | +Inf (0.89, 0) | +0.25 (4.21, 3.54) | +Inf (0.89, 0) | +Inf (0.89, 0) | -0.95 (4.21, 8.13) | +Inf (0.88, 0) | NaN (0, 0) | -1.2 (3.54, 8.13) |
| DMv3 | all | -0.13 (3.17, 3.48) | -0.58 (2.32, 3.48) | -0.43 (4.97, 6.7) | **+0.95 (3.17, 1.65)** | **+1.05 (2.32, 1.12)** | **+0.72 (4.97, 3.01)** | **+1.08 (3.48, 1.65)** | **+1.63 (3.48, 1.12)** | **+1.15 (6.7, 3.01)** |
| DMv3 | high_quality | +0.06 (4.1, 3.94) | -0.36 (3.06, 3.94) | -0.38 (5.93, 7.73) | **+1.22 (4.1, 1.76)** | **+1.9 (3.06, 0.82)** | **+1.01 (5.93, 2.94)** | +1.16 (3.94, 1.76) | **+2.26 (3.94, 0.82)** | **+1.4 (7.73, 2.94)** |
| DMv3 | cap | -0.13 (6.1, 6.68) | -0.46 (4.85, 6.68) | -0.46 (8.19, 11.27) | **+1.02 (6.1, 3.01)** | **+1.69 (4.85, 1.5)** | +0.57 (8.19, 5.51) | +1.15 (6.68, 3.01) | **+2.15 (6.68, 1.5)** | **+1.03 (11.27, 5.51)** |
| DMv3 | EPD | +0.45 (7.24, 5.31) | -0.14 (4.81, 5.31) | +0.41 (9.44, 7.08) | +1.57 (7.24, 2.44) | +1.56 (4.81, 1.63) | +1.95 (9.44, 2.44) | +1.12 (5.31, 2.44) | +1.71 (5.31, 1.63) | +1.54 (7.08, 2.44) |
| DMv4 | all | **-0.72 (3.39, 5.58)** | -0.27 (3.39, 4.09) | -0.28 (5.05, 6.14) | **+1.47 (3.39, 1.23)** | **+1.6 (3.39, 1.12)** | **+0.94 (5.05, 2.63)** | **+2.18 (5.58, 1.23)** | **+1.87 (4.09, 1.12)** | **+1.22 (6.14, 2.63)** |
| DMv4 | high_quality | -0.13 (4.28, 4.7) | +0.18 (4.28, 3.79) | -0.02 (6.26, 6.36) | **+1.86 (4.28, 1.18)** | **+1.86 (4.28, 1.18)** | **+1.03 (6.26, 3.06)** | **+2 (4.7, 1.18)** | **+1.69 (3.79, 1.18)** | +1.06 (6.36, 3.06) |
| DMv4 | cap | -0.42 (6.54, 8.77) | +0.38 (6.54, 5.01) | +0.23 (8.56, 7.31) | **+1.39 (6.54, 2.5)** | **+1.2 (6.54, 2.84)** | +0.77 (8.56, 5.01) | **+1.81 (8.77, 2.5)** | +0.82 (5.01, 2.84) | +0.54 (7.31, 5.01) |
| DMv4 | EPD | -0.03 (6.94, 7.08) | +0.65 (6.94, 4.42) | +0.4 (11.69, 8.85) | +2.09 (6.94, 1.63) | +3.09 (6.94, 0.81) | **+2.26 (11.69, 2.44)** | +2.12 (7.08, 1.63) | +2.44 (4.42, 0.81) | +1.86 (8.85, 2.44) |

| | | | | | | | | | | |
|---|---|---|---|---|---|---|---|---|---|---|
| DMv5 | all | +0.53 (1.41, 0.97) | -0.68 (1.41, 2.25) | **-0.7 (2.24, 3.63)** | +0.68 (1.41, 0.88) | +0.87 (1.41, 0.77) | +0.61 (2.24, 1.47) | +0.15 (0.97, 0.88) | **+1.55 (2.25, 0.77)** | **+1.3 (3.63, 1.47)** |
| DMv5 | high_quality | +0.62 (1.63, 1.06) | -0.57 (1.63, 2.42) | -0.4 (2.65, 3.48) | +0.48 (1.63, 1.18) | +0.8 (1.63, 0.94) | +0.49 (2.65, 1.88) | -0.15 (1.06, 1.18) | +1.37 (2.42, 0.94) | +0.89 (3.48, 1.88) |
| DMv5 | cap | +1.07 (2.62, 1.25) | -0.44 (2.62, 3.55) | -0.51 (3.8, 5.43) | +1.39 (2.62, 1) | +1.39 (2.62, 1) | +0.81 (3.8, 2.17) | +0.32 (1.25, 1) | +1.83 (3.55, 1) | +1.32 (5.43, 2.17) |
| DMv5 | EPD | +1.86 (3.2, 0.88) | +0.86 (3.2, 1.77) | +0.69 (5.7, 3.54) | +Inf (3.2, 0) | +Inf (3.2, 0) | +Inf (5.7, 0) | +Inf (0.88, 0) | +Inf (1.77, 0) | +Inf (3.54, 0) |
| GAGA/NDM1 | all | **-1.21 (1.23, 2.86)** | **-1.15 (1.66, 3.68)** | **-1.19 (1.96, 4.45)** | **-1.98 (1.23, 4.87)** | **-1.55 (1.66, 4.87)** | **-1.6 (1.96, 5.92)** | **-0.77 (2.86, 4.87)** | -0.4 (3.68, 4.87) | -0.41 (4.45, 5.92) |
| GAGA/NDM1 | high_quality | -0.93 (1.43, 2.73) | -0.94 (1.9, 3.64) | **-1.06 (2.18, 4.55)** | **-1.92 (1.43, 5.41)** | **-1.51 (1.9, 5.41)** | **-1.57 (2.18, 6.46)** | -0.99 (2.73, 5.41) | -0.57 (3.64, 5.41) | -0.51 (4.55, 6.46) |
| GAGA/NDM1 | cap | -0.67 (2.23, 3.55) | -0.48 (2.85, 3.97) | -0.45 (3.2, 4.38) | **-1.35 (2.23, 5.68)** | **-0.99 (2.85, 5.68)** | **-1.23 (3.2, 7.51)** | -0.68 (3.55, 5.68) | -0.52 (3.97, 5.68) | -0.78 (4.38, 7.51) |
| GAGA/NDM1 | EPD | -1.13 (2.02, 4.42) | -0.99 (2.67, 5.31) | -0.7 (3.26, 5.31) | **-2.39 (2.02, 10.57)** | **-1.98 (2.67, 10.57)** | **-1.9 (3.26, 12.2)** | -1.26 (4.42, 10.57) | -0.99 (5.31, 10.57) | -1.2 (5.31, 12.2) |
| NDM2 | all | **-0.71 (2.92, 4.76)** | **-0.71 (2.92, 4.76)** | -0.33 (3.16, 3.99) | **-1.05 (2.92, 6.03)** | **-0.56 (2.92, 4.31)** | **-0.76 (3.16, 5.36)** | -0.34 (4.76, 6.03) | +0.14 (4.76, 4.31) | -0.43 (3.99, 5.36) |
| NDM2 | high_quality | **-0.87 (3.33, 6.06)** | **-0.87 (3.33, 6.06)** | -0.52 (3.37, 4.85) | **-1.11 (3.33, 7.17)** | -0.7 (3.33, 5.41) | **-0.83 (3.37, 5.99)** | -0.24 (6.06, 7.17) | +0.17 (6.06, 5.41) | -0.31 (4.85, 5.99) |
| NDM2 | cap | +0.06 (4.34, 4.18) | +0.06 (4.34, 4.18) | +0.9 (3.9, 2.09) | **-0.97 (4.34, 8.51)** | -0.55 (4.34, 6.34) | -0.66 (3.9, 6.18) | -1.03 (4.18, 8.51) | -0.6 (4.18, 6.34) | **-1.56 (2.09, 6.18)** |
| NDM2 | EPD | -0.38 (6.11, 7.96) | -0.38 (6.11, 7.96) | -0.95 (5.04, 9.73) | **-1.26 (6.11, 14.63)** | -1.09 (6.11, 13.01) | -1.07 (5.04, 10.57) | -0.88 (7.96, 14.63) | -0.71 (7.96, 13.01) | -0.12 (9.73, 10.57) |
| NDM3 | all | +0.2 (1.11, 0.97) | -0.46 (1.11, 1.53) | -0.58 (1.6, 2.4) | **+Inf (1.11, 0)** | -0.05 (1.11, 1.16) | -0.46 (1.6, 2.21) | **+Inf (0.97, 0)** | +0.41 (1.53, 1.16) | +0.12 (2.4, 2.21) |
| NDM3 | high_quality | +0.05 (1.25, 1.21) | -0.65 (1.25, 1.97) | -0.8 (1.66, 2.88) | **+Inf (1.25, 0)** | +1.09 (1.25, 0.59) | -0.09 (1.66, 1.76) | **+Inf (1.21, 0)** | +1.75 (1.97, 0.59) | +0.71 (2.88, 1.76) |
| NDM3 | cap | +0.27 (1.51, 1.25) | -0.47 (1.51, 2.09) | -0.57 (1.97, 2.92) | **+Inf (1.51, 0)** | +0.01 (1.51, 1.5) | +0.39 (1.97, 1.5) | +Inf (1.25, 0) | +0.47 (2.09, 1.5) | +0.96 (2.92, 1.5) |
| NDM3 | EPD | -0.81 (1.01, 1.77) | -1.4 (1.01, 2.65) | -1.27 (1.84, 4.42) | +Inf (1.01, 0) | +Inf (1.01, 0) | +0.18 (1.84, 1.63) | +Inf (1.77, 0) | +Inf (2.65, 0) | +1.44 (4.42, 1.63) |
| DRE/NDM4 | all | **-0.77 (8.34, 14.22)** | -0.38 (6.24, 8.13) | **-0.48 (12.68, 17.7)** | **+0.86 (8.34, 4.59)** | **+1.12 (6.24, 2.87)** | **+0.81 (12.68, 7.25)** | **+1.63 (14.22, 4.59)** | **+1.5 (8.13, 2.87)** | **+1.29 (17.7, 7.25)** |
| DRE/NDM4 | high_quality | **-0.53 (10.39, 15)** | -0.16 (7.71, 8.64) | -0.25 (15.42, 18.33) | **+0.82 (10.39, 5.88)** | **+1.13 (7.71, 3.53)** | **+0.83 (15.42, 8.7)** | **+1.35 (15, 5.88)** | **+1.29 (8.64, 3.53)** | **+1.08 (18.33, 8.7)** |
| DRE/NDM4 | cap | -0.33 (14.92, 18.79) | -0.23 (10.69, 12.53) | -0.17 (20.23, 22.76) | **+1.12 (14.92, 6.84)** | **+0.96 (10.69, 5.51)** | **+0.79 (20.23, 11.69)** | **+1.46 (18.79, 6.84)** | **+1.19 (12.53, 5.51)** | **+0.96 (22.76, 11.69)** |
| DRE/NDM4 | EPD | -0.54 (13.41, 19.47) | -0.11 (11.45, 12.39) | -0.03 (21.66, 22.12) | +1.46 (13.41, 4.88) | **+2.82 (11.45, 1.63)** | **+1.28 (21.66, 8.94)** | **+2 (19.47, 4.88)** | +2.93 (12.39, 1.63) | +1.31 (22.12, 8.94) |
| E-box/NDM5 | all | +0.29 (5.44, 4.45) | +0.19 (5.44, 4.76) | +0.08 (10.19, 9.62) | **+Inf (5.44, 0)** | **+0.94 (5.44, 2.84)** | **+0.57 (10.19, 6.87)** | **+Inf (4.45, 0)** | **+0.75 (4.76, 2.84)** | **+0.49 (9.62, 6.87)** |
| E-box/NDM5 | high_quality | +0.59 (6.38, 4.24) | +0.49 (6.38, 4.55) | +0.29 (11.67, 9.55) | **+Inf (6.38, 0)** | **+1.18 (6.38, 2.82)** | **+0.57 (11.67, 7.87)** | **+Inf (4.24, 0)** | +0.69 (4.55, 2.82) | +0.28 (9.55, 7.87) |
| E-box/NDM5 | cap | +0.62 (8.03, 5.22) | +0.68 (8.03, 5.01) | +0.47 (13.34, 9.6) | **+Inf (8.03, 0)** | **+1.19 (8.03, 3.51)** | **+0.62 (13.34, 8.68)** | **+Inf (5.22, 0)** | +0.52 (5.01, 3.51) | +0.15 (9.6, 8.68) |
| E-box/NDM5 | EPD | +0.44 (4.81, 3.54) | +0.44 (4.81, 3.54) | +0.26 (9.55, 7.96) | +Inf (4.81, 0) | +0.98 (4.81, 2.44) | +0.38 (9.55, 7.32) | +Inf (3.54, 0) | +0.54 (3.54, 2.44) | +0.12 (7.96, 7.32) |
| None | all | **+0.17 (61.81, 54.78)** | **+0.13 (63.38, 58.01)** | **+0.21 (49.51, 42.76)** | **-0.18 (61.81, 69.9)** | **-0.12 (63.38, 68.82)** | **-0.16 (49.51, 55.36)** | **-0.35 (54.78, 69.9)** | **-0.25 (58.01, 68.82)** | **-0.37 (42.76, 55.36)** |
| None | high_quality | +0.02 (56.09, 55.3) | +0.02 (58.13, 57.42) | +0.05 (43.52, 42.12) | **-0.26 (56.09, 66.98)** | **-0.2 (58.13, 66.75)** | **-0.27 (43.52, 52.53)** | **-0.28 (55.3, 66.98)** | **-0.22 (57.42, 66.75)** | **-0.32 (42.12, 52.53)** |
| None | cap | -0.12 (40.92, 44.47) | -0.14 (44.26, 48.64) | -0.13 (30.71, 33.61) | **-0.5 (40.92, 57.76)** | **-0.37 (44.26, 57.1)** | **-0.53 (30.71, 44.41)** | **-0.38 (44.47, 57.76)** | -0.23 (48.64, 57.1) | **-0.4 (33.61, 44.41)** |
| None | EPD | +0.14 (42.91, 38.94) | +0.01 (44.63, 44.25) | -0.33 (26.82, 33.63) | -0.06 (42.91, 44.72) | -0.08 (44.63, 47.15) | -0.2 (26.82, 30.89) | -0.2 (38.94, 44.72) | -0.09 (44.25, 47.15) | +0.12 (33.63, 30.89) |

[a]The position requirements were calculated based on the distribution of each motif in each of the three promoter classes (UPs/FAPs/DAPs) of fly genome; see Methods

[b]The position requirements were calculated based on the distribution of each motif in all promoters of fly genome; see Methods

[c]The position requirements were taken from FitzGerald et al. (2006).

[d]The promoter set contains all promoters from *Drosophila* genome annotation release 5.5.

[e]The promoter set contains only high quality promoters in fly genome; see Methods

[f]The promoter set contains only cap-supported promoters in fly genome; see Methods

[g]The promoter set contains only *Drosophila* promoters from EPD; see Methods

[h]The numbers are in bold when the presence of the motif differ significantly between two promoter classes. The first number in parenthesis is the percentage of promoters in the first class containing the motif. The second number in parenthesis is the percentage of promoters in the second class containing the motif.

**Table S3: The $log_2$ relative probability of fly promoter motif present in any two of the three promoter classes**

| Motif | | UPs vs. FAPs | | UPs vs. DAPs | | FAPs vs. DAPs | |
|---|---|---|---|---|---|---|---|
| | | regular expression[a] | PWM[b] | regular expression | PWM | regular expression | PWM |
| TATA/DMp1 | all[c] | **+2.36 (5.77, 1.13)**[f] | **+2.43 (8.82, 1.64)** | **+2.61 (5.77, 0.95)** | **+2.42 (8.82, 1.65)** | +0.25 (1.13, 0.95) | -0.01 (1.64, 1.65) |
| TATA/DMp1 | high_quality[d] | **+2.22 (5.65, 1.21)** | **+2.24 (8.58, 1.82)** | **+2.27 (5.65, 1.18)** | **+2.73 (8.58, 1.29)** | +0.04 (1.21, 1.18) | +0.49 (1.82, 1.29) |
| TATA/DMp1 | cap[e] | **+3.12 (7.28, 0.84)** | **+2.44 (10.21, 1.88)** | **+2.13 (7.28, 1.67)** | **+1.85 (10.21, 2.84)** | -1 (0.84, 1.67) | -0.6 (1.88, 2.84) |
| INR/DMp2 | all | -0.23 (10.22, 11.97) | **-0.24 (14.25, 16.83)** | **-0.3 (10.22, 12.58)** | **-0.36 (14.25, 18.29)** | -0.07 (11.97, 12.58) | -0.12 (16.83, 18.29) |
| INR/DMp2 | high_quality | +0.19 (11.42, 10) | 0 (16.06, 16.06) | -0.17 (11.42, 12.81) | -0.23 (16.06, 18.8) | -0.36 (10, 12.81) | -0.23 (16.06, 18.8) |
| INR/DMp2 | cap | -0.01 (14.71, 14.82) | -0.1 (19.07, 20.46) | -0.28 (14.71, 17.86) | **-0.35 (19.07, 24.37)** | -0.27 (14.82, 17.86) | -0.25 (20.46, 24.37) |
| INR1/DMp3 | all | **-1.45 (0.52, 1.43)** | —[g] | -0.49 (0.52, 0.74) | — | +0.96 (1.43, 0.74) | — |
| INR1/DMp3 | high_quality | -1.4 (0.63, 1.67) | — | -0.38 (0.63, 0.82) | — | +1.02 (1.67, 0.82) | — |
| INR1/DMp3 | cap | +0.09 (1.11, 1.04) | — | -0.58 (1.11, 1.67) | — | -0.68 (1.04, 1.67) | — |
| DPE/DMp4 | all | -0.41 (0.69, 0.92) | **-0.63 (2.68, 4.14)** | -0.55 (0.69, 1.02) | -0.36 (2.68, 3.43) | -0.14 (0.92, 1.02) | +0.27 (4.14, 3.43) |
| DPE/DMp4 | high_quality | +0.12 (0.82, 0.76) | -0.07 (3.33, 3.48) | -0.19 (0.82, 0.94) | -0.13 (3.33, 3.64) | -0.31 (0.76, 0.94) | -0.06 (3.48, 3.64) |
| DPE/DMp4 | cap | -0.02 (1.44, 1.46) | -0.05 (5.24, 5.43) | -0.06 (1.44, 1.5) | -0.03 (5.24, 5.34) | -0.04 (1.46, 1.5) | +0.02 (5.43, 5.34) |
| DPE1/DMp5 | all | **-1.45 (0.39, 1.07)** | — | -1.03 (0.39, 0.81) | — | +0.41 (1.07, 0.81) | — |
| DPE1/DMp5 | high_quality | -1.35 (0.48, 1.21) | — | -1.44 (0.48, 1.29) | — | -0.09 (1.21, 1.29) | — |
| DPE1/DMp5 | cap | +0.12 (0.9, 0.84) | — | +0.12 (0.9, 0.83) | — | 0 (0.84, 0.83) | — |
| DMv1 | all | -0.65 (1.56, 2.46) | — | **+1.16 (1.56, 0.7)** | — | **+1.81 (2.46, 0.7)** | — |
| DMv1 | high_quality | -0.38 (1.86, 2.42) | — | +1.66 (1.86, 0.59) | — | +2.04 (2.42, 0.59) | — |
| DMv1 | cap | -0.18 (2.57, 2.92) | — | +0.78 (2.57, 1.5) | — | +0.96 (2.92, 1.5) | — |
| DMv2 | all | -0.03 (0.85, 0.87) | -0.45 (1.84, 2.51) | **+Inf (0.85, 0)** | **+Inf (1.84, 0)** | **+Inf (0.87, 0)** | **+Inf (2.51, 0)** |
| DMv2 | high_quality | +0.07 (0.95, 0.91) | -0.11 (2.11, 2.27) | **+Inf (0.95, 0)** | **+Inf (2.11, 0)** | +Inf (0.91, 0) | **+Inf (2.27, 0)** |
| DMv2 | cap | +1.44 (1.14, 0.42) | +0.29 (2.81, 2.3) | +Inf (1.14, 0) | **+Inf (2.81, 0)** | +Inf (0.42, 0) | **+Inf (2.3, 0)** |
| DMv3 | all | -0.13 (3.17, 3.48) | -0.14 (6.57, 7.26) | **+0.95 (3.17, 1.65)** | **+0.81 (6.57, 3.75)** | **+1.08 (3.48, 1.65)** | **+0.95 (7.26, 3.75)** |
| DMv3 | high_quality | +0.06 (4.1, 3.94) | +0.3 (12.46, 10.15) | **+1.22 (4.1, 1.76)** | **+0.82 (12.46, 7.05)** | +1.16 (3.94, 1.76) | +0.53 (10.15, 7.05) |
| DMv3 | cap | -0.13 (6.1, 6.68) | +0.24 (17.51, 14.82) | **+1.02 (6.1, 3.01)** | **+0.83 (17.51, 9.85)** | +1.15 (6.68, 3.01) | +0.59 (14.82, 9.85) |
| DMv4 | all | **-0.72 (3.39, 5.58)** | **-0.66 (8.15, 12.89)** | **+1.47 (3.39, 1.23)** | **+0.94 (8.15, 4.24)** | **+2.18 (5.58, 1.23)** | **+1.6 (12.89, 4.24)** |
| DMv4 | high_quality | -0.13 (4.28, 4.7) | -0.32 (10.05, 12.58) | **+1.86 (4.28, 1.18)** | **+1.1 (10.05, 4.7)** | **+2 (4.7, 1.18)** | **+1.42 (12.58, 4.7)** |
| DMv4 | cap | -0.42 (6.54, 8.77) | -0.22 (15.03, 17.54) | **+1.39 (6.54, 2.5)** | **+1.1 (15.03, 7.01)** | **+1.81 (8.77, 2.5)** | **+1.32 (17.54, 7.01)** |
| DMv5 | all | +0.53 (1.41, 0.97) | **+0.76 (4.95, 2.92)** | +0.68 (1.41, 0.88) | +0.4 (4.95, 3.75) | +0.15 (0.97, 0.88) | -0.36 (2.92, 3.75) |
| DMv5 | high_quality | +0.62 (1.63, 1.06) | +0.91 (5.68, 3.03) | +0.48 (1.63, 1.18) | +0.51 (5.68, 4) | -0.15 (1.06, 1.18) | -0.4 (3.03, 4) |
| DMv5 | cap | +1.07 (2.62, 1.25) | **+1.23 (7.84, 3.34)** | +1.39 (2.62, 1) | **+0.91 (7.84, 4.17)** | +0.32 (1.25, 1) | -0.32 (3.34, 4.17) |
| GAGA/NDM1 | all | **-1.21 (1.23, 2.86)** | — | **-1.98 (1.23, 4.87)** | — | **-0.77 (2.86, 4.87)** | — |
| GAGA/NDM1 | high_quality | -0.93 (1.43, 2.73) | — | **-1.92 (1.43, 5.41)** | — | -0.99 (2.73, 5.41) | — |
| GAGA/NDM1 | cap | -0.67 (2.23, 3.55) | — | **-1.35 (2.23, 5.68)** | — | -0.68 (3.55, 5.68) | — |
| NDM2 | all | **-0.71 (2.92, 4.76)** | — | **-1.05 (2.92, 6.03)** | — | -0.34 (4.76, 6.03) | — |
| NDM2 | high_quality | **-0.87 (3.33, 6.06)** | — | **-1.11 (3.33, 7.17)** | — | -0.24 (6.06, 7.17) | — |
| NDM2 | cap | +0.06 (4.34, 4.18) | — | **-0.97 (4.34, 8.51)** | — | -1.03 (4.18, 8.51) | — |
| NDM3 | all | +0.2 (1.11, 0.97) | — | **+Inf (1.11, 0)** | — | **+Inf (0.97, 0)** | — |

| | | | | | | | |
|---|---|---|---|---|---|---|---|
| NDM3 | high_quality | +0.05 (1.25, 1.21) | — | **+Inf (1.25, 0)** | — | **+Inf (1.21, 0)** | — |
| NDM3 | cap | +0.27 (1.51, 1.25) | — | **+Inf (1.51, 0)** | — | +Inf (1.25, 0) | — |
| DRE/NDM4 | all | **-0.77 (8.34, 14.22)** | **-0.65 (12.21, 19.18)** | **+0.86 (8.34, 4.59)** | **+0.79 (12.21, 7.04)** | **+1.63 (14.22, 4.59)** | **+1.45 (19.18, 7.04)** |
| DRE/NDM4 | high_quality | **-0.53 (10.39, 15)** | **-0.44 (14.67, 19.85)** | **+0.82 (10.39, 5.88)** | **+0.77 (14.67, 8.58)** | **+1.35 (15, 5.88)** | **+1.21 (19.85, 8.58)** |
| DRE/NDM4 | cap | -0.33 (14.92, 18.79) | -0.33 (19.9, 25.05) | **+1.12 (14.92, 6.84)** | **+1.25 (19.9, 8.35)** | **+1.46 (18.79, 6.84)** | **+1.59 (25.05, 8.35)** |
| E-box/NDM5 | all | +0.29 (5.44, 4.45) | +0.24 (9.73, 8.24) | **+Inf (5.44, 0)** | **+Inf (9.73, 0)** | **+Inf (4.45, 0)** | **+Inf (8.24, 0)** |
| E-box/NDM5 | high_quality | +0.59 (6.38, 4.24) | +0.37 (10.58, 8.18) | **+Inf (6.38, 0)** | **+Inf (10.58, 0)** | **+Inf (4.24, 0)** | **+Inf (8.18, 0)** |
| E-box/NDM5 | cap | +0.62 (8.03, 5.22) | +0.31 (12.43, 10.02) | **+Inf (8.03, 0)** | **+Inf (12.43, 0)** | **+Inf (5.22, 0)** | **+Inf (10.02, 0)** |
| None | all | **+0.17 (61.81, 54.78)** | **+0.14 (48.74, 44.09)** | **-0.18 (61.81, 69.9)** | **-0.41 (48.74, 64.65)** | **-0.35 (54.78, 69.9)** | **-0.55 (44.09, 64.65)** |
| None | high_quality | +0.02 (56.09, 55.3) | -0.12 (40.8, 44.24) | **-0.26 (56.09, 66.98)** | **-0.58 (40.8, 61.1)** | **-0.28 (55.3, 66.98)** | **-0.47 (44.24, 61.1)** |
| None | cap | -0.12 (40.92, 44.47) | -0.19 (25.93, 29.65) | **-0.5 (40.92, 57.76)** | **-0.97 (25.93, 50.75)** | **-0.38 (44.47, 57.76)** | **-0.78 (29.65, 50.75)** |

[a]The motifs were searched using the consensus sequences given by FitzGerald *et al.* (2006); see Methods

[b]The motifs were searched using PWMs given by Ohler *et al.* (2002); see Methods

[c]The promoter set contains all promoters from *Drosophila* genome annotation release 5.5.

[d]The promoter set contains only high quality promoters in fly genome; see Methods

[e]The promoter set contains only cap-supported promoters in fly genome; see Methods

[f]The numbers are in bold when the presence of the motif differ significantly between two promoter classes. The first number in parenthesis is the percentage of promoters in the first class containing the motif. The second number in parenthesis is the percentage of promoters in the second class containing the motif.

[g]The corresponding motif was not available in Ohler *et al.* (2002).

Table S4: The eight human promoter motifs[a]

| Motif | Consensus sequence[b] | Strand[c] | Valid Range[d] |
|---|---|---|---|
| CCAAT | RRCCAATSR | both | -180 to -21 |
| SP1 | CCCGCCC | both | -140 to -1 |
| CLUS1 | TCTCGCGA | both | -160 to 20 |
| USF | TCACGTGR | both | -100 to 20 |
| CREB | TGAYGTCA | both | -120 to 20 |
| TATA | TATAWRD | + | -60 to -1 |
| NRF-1 | CGCVTGCG | both | -100 to 20 |
| ETS | SCGGAARY | both | -140 to 20 |

[a]As given by FitzGerald *et al*. (2004)

[b]R=G, A; W=A, T; Y=T, C; K=G, T; M=A, C; S=G, C; N=A, T, G, C

[c]+, coding strand; both, either strand

[d]relative to the TSS; see Methods
