# Supplementary material for: Complex organizational structure of the genome revealed by genome-wide analysis of single and alternative promoters in Drosophila melanogaster
Source: BMC Genomics. 2009 Jan 7;10:9. doi: 10.1186/1471-2164-10-9 (PMC2631479; doi:10.1186/1471-2164-10-9)
Supplement: Additional file 6 — Figure S3. Motif dissimilarity distributions between alternative promoters of the same gene and between neighboring unique promoters. [file 1471-2164-10-9-S6.pdf]

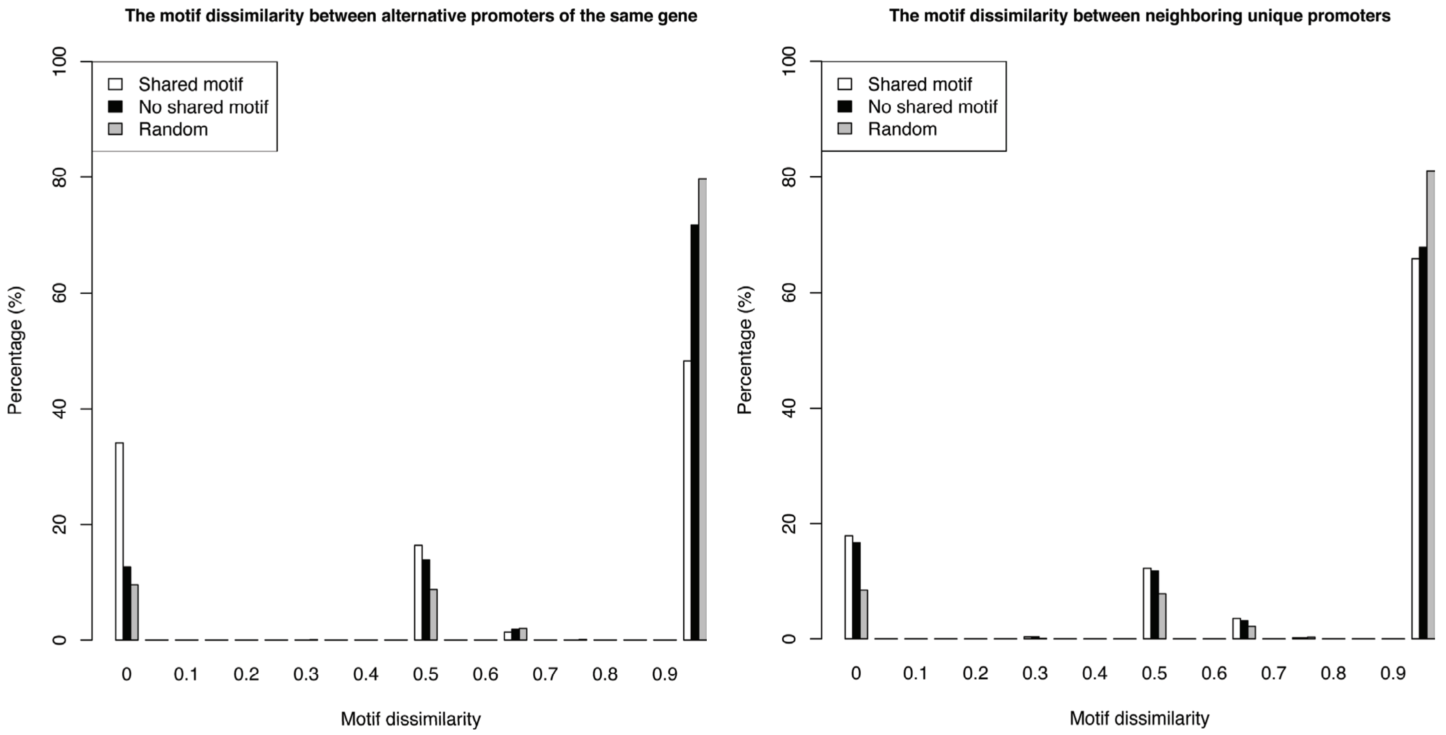

Figure S3: Motif dissimilarity distribution between alternative promoters of the same gene, and between neighboring unique promoters. See text for details.
