# Supplementary material for: Complex organizational structure of the genome revealed by genome-wide analysis of single and alternative promoters in Drosophila melanogaster
Source: BMC Genomics. 2009 Jan 7;10:9. doi: 10.1186/1471-2164-10-9 (PMC2631479; doi:10.1186/1471-2164-10-9)
Supplement: Additional file 8 — Figure S4. Distribution of the number of mapped motifs in individual promoters. [file 1471-2164-10-9-S8.pdf]

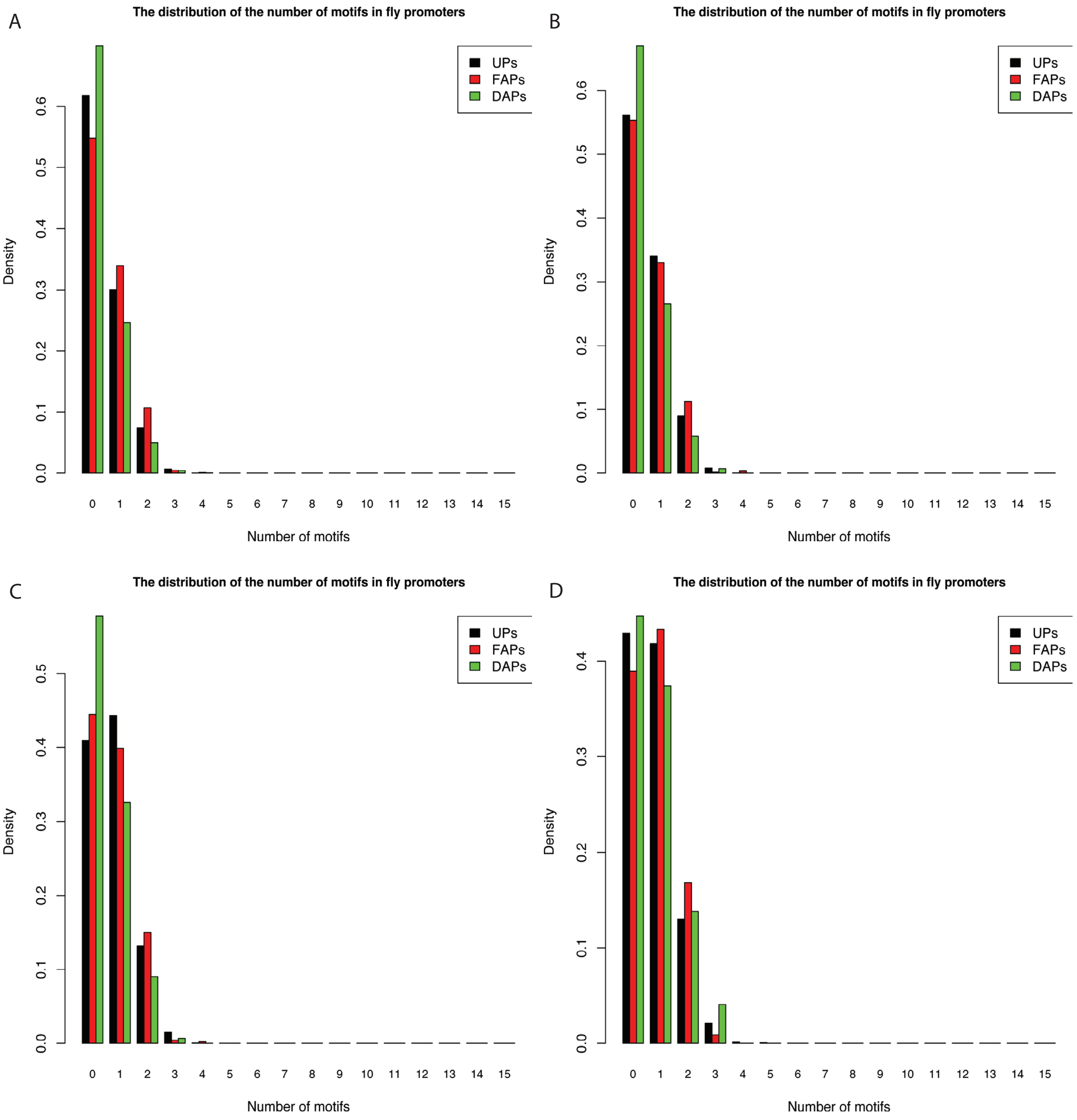

Figure S4: Distribution of the numbers of known motifs mapped to individual promoters. (A) All fly promoters; (B) "high quality" fly promoters; (C) cap-supported fly promoters; (D) fly promoters from the EPD; (E) 4506 human promoters from Baek et al. (2007).

E

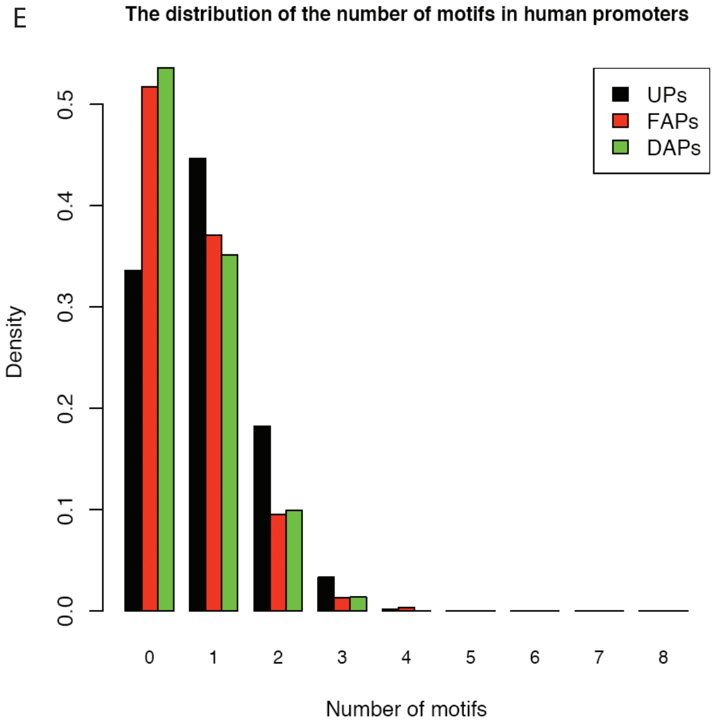

Figure S4: Distribution of the numbers of known motifs mapped to individual promoters. (A) All fly promoters; (B) "high quality" fly promoters; (C) cap-supported fly promoters; (D) fly promoters from the EPD; (E) 4506 human promoters from Baek et al. (2007).
