# Supplementary material for: Complex organizational structure of the genome revealed by genome-wide analysis of single and alternative promoters in Drosophila melanogaster
Source: BMC Genomics. 2009 Jan 7;10:9. doi: 10.1186/1471-2164-10-9 (PMC2631479; doi:10.1186/1471-2164-10-9)
Supplement: Additional file 9 — Figure S5. Histogram of distances between alternative TSSs for the same gene in the fly genome when all promoters in the genome (A) or only cap-supported promoters (B) were considered. [file 1471-2164-10-9-S9.pdf]

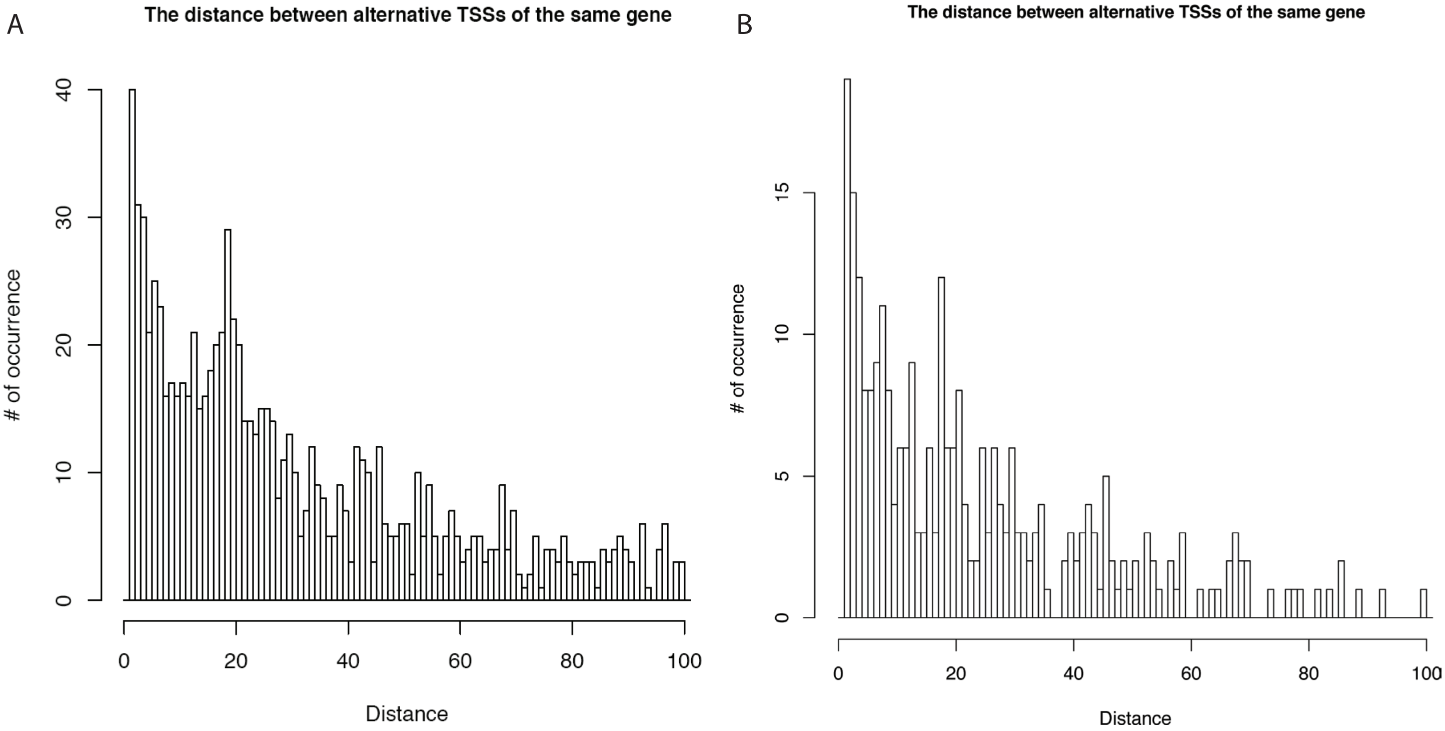

Figure S5: Histogram of distances between alternative TSSs for the same gene in the fly genome when (A) all promoters are considered and (B) when only the "cap-supported" promoters are considered. Alternative TSSs whose distances are shorter than a cutoff determined by the position of the second peak in the distribution were considered to derive from the same promoter.
